# Role of long non-coding RNA in regulatory network response to *Candidatus* Liberibacter asiaticus in citrus

**DOI:** 10.3389/fpls.2023.1090711

**Published:** 2023-02-20

**Authors:** Xiaokang Zhuo, Qibin Yu, Riccardo Russo, Yi Zhang, Xu Wei, Yuanzhi Zimmy Wang, Paige Marie Holden, Fred G. Gmitter

**Affiliations:** Citrus Research and Education Center, Institute of Food and Agricultural Sciences, University of Florida, Lake Alfred, FL, United States

**Keywords:** long non-coding RNA, citrus Huanglongbing, weighted gene co-expression network analysis, miRNA5021, plant immunity

## Abstract

Long non-coding RNAs (lncRNAs) serve as crucial regulators in plant response to various diseases, while none have been systematically identified and characterized in response to citrus Huanglongbing (HLB) caused by *Candidatus* Liberibacter asiaticus (*C*Las) bacteria. Here, we comprehensively investigated the transcriptional and regulatory dynamics of the lncRNAs in response to *C*L*as*. Samples were collected from leaf midribs of *C*Las- and mock-inoculated HLB-tolerant rough lemon (*Citrus jambhiri*) and HLB-sensitive sweet orange (*C. sinensis*) at week 0, 7, 17, and 34 following inoculation using *C*Las+ budwood of three biological replicates in the greenhouse. A total of 8,742 lncRNAs, including 2,529 novel lncRNAs, were identified from RNA-seq data with rRNA-removed from strand-specific libraries. Genomic variation analyses of conserved lncRNAs from 38 citrus accessions showed that 26 single nucleotide polymorphisms (SNPs) were significantly correlated with HLB. In addition, lncRNA-mRNA weighted gene co-expression network analysis (WGCNA) showed a significant module correlated with *C*Las-inoculation in rough lemon. Notably, the most significant *LNC_28805* and multiple co-expressed genes related to plant defense in the module were targeted by *miRNA5021*, suggesting that *LNC28805* might compete with endogenous *miR5021* to maintain the homeostasis of immune gene expression levels. Candidate *WRKY33* and *SYP121* genes targeted by *miRNA5021* were identified as two key hub genes interacting with bacteria pathogen response genes based on the prediction of protein-protein interaction (PPI) network. These two genes were also found within HLB-associated QTL in linkage group 6. Overall, our findings provide a reference for a better understanding of the role of lncRNAs involved in citrus HLB regulation.

## 1 Introduction

Transcripts with a length of more than 200 nt and lower protein-coding potential are operationally termed long non-coding RNAs (lncRNAs), which are widespread non-coding RNAs (ncRNAs) in eukaryotes. In animals and plants, lncRNA can function as important and versatile regulators in a variety of cellular and biological processes (Kim and Sung, 2012). Biochemical experiments and genetics studies have demonstrated that lncRNAs are associated with chromatin modification (Rinn and Chang, 2012), mRNA splicing (Bardou et al., 2014), transcriptional gene silencing (Wierzbicki, 2012), and posttranscriptional gene regulation (Yoon et al., 2013). Studies indicate that lncRNAs can help the host to prevent pathogen replication or be used by pathogens to promote pathogen proliferation (Li et al., 2016; Zaynab et al., 2018; Shirahama et al., 2020). In tomato, yellow leaf curl virus intergenic siRNAs target a host long noncoding RNA to modulate disease symptoms (Yang et al., 2019); also, tomato lncRNA23468 functions as a competing endogenous miR482b to enhance the defense against *Phytophthora infestans* (Jiang et al., 2019). It was also found that lncRNAs function as precursors of miRNAs having stable hairpin structures in wheat (Xin et al., 2011). In a word, lncRNAs are involved in pathogenic infection by acting as miRNA targets, miRNA precursors, or endogenous target mimics (eTMs) to regulate the expression of their target genes (Shirahama et al., 2020; Song et al., 2021).

Recent studies have revealed a set of the important regulatory functions of lncRNAs in response to pathogen infection. Transcriptome analyses revealed that a large number of lncRNAs were differentially expressed in response to pathogen infection in plants. For instance, *lncRNA16397* was involved in resistance to *P. infestans* infection by co-expressing glutaredoxin in tomato (Cui et al., 2017). In melon, lncRNAs function as miRNA precursors and are involved in the response of powdery mildew fungi (Gao et al., 2020). Also, lncRNAs are involved in the response of *Arabidopsis thaliana* to *Fusarium oxysporum* infection (Zhu et al., 2014) and cotton to *Verticillium dahlia* infection (Zhang et al., 2018). In addition, a study also found that interaction between Sl-lncRNA15492 and Sl-miR482a can affect *Solanum lycopersicum* immunity against *P. infestans* (Jiang et al., 2020). Thus, lncRNAs are important components of regulated networks in response to pathogen infection. Although many lncRNAs have been identified from transcriptome data in diverse plant species, most of them are not well characterized.

Huanglongbing (HLB), a disease caused by the phloem-limited bacterium *Candidatus* Liberibacter asiaticus (*C*Las), is the most prevalent and destructive citrus epidemic. It has devastated the citrus industry in Florida and is threatening the global citrus industries (Bové, 2006; Graham et al., 2020). Thus far, citrus HLB has not been controlled effectively, and some research directions are precluded because of the challenge of *C*Las’ unculturable and phloem-limited nature. HLB and its vector, Asian citrus psyllid (ACP, Diaphorina citri) is still rapidly spreading in citrus-producing areas, which leads to billions of dollars in annual economic loss (Alvarez et al., 2016; Wang, 2019; Monzó and Stansly, 2020). Current strategies for insecticide and antibiotics application are limited and unsustainable. One of the most effective and eco-friendly strategies is strengthening host plant defense and immunity. Usually, the plant innate immune response can be triggered when they are infected by the pathogen (Nobori and Tsuda, 2019). In citrus, *C*Las-triggered plant immune responses are delayed 5–9 weeks after inoculation (Albrecht and Bowman, 2008; Yu et al., 2017). Traditional molecular biology, genetic, and multi-omics analyses also incompletely revealed the nature of pathogenesis of citrus HLB (da Graça et al., 2016; Wang, 2019). A study indicated that *Citrus tristeza virus* (CTV) can produce LMT1 lncRNA to suppress salicylic acid (SA) accumulation and mitigate reactive oxygen species (ROS) accumulation (Kang et al., 2019). These cases related to lncRNA involved in plant disease regulation bring us a promising direction to explore the role of lncRNA against citrus HLB disease (Jiang et al., 2020; Hong et al., 2022; Sharma et al., 2022). Thus far, no studies have explored such roles and characteristics in citrus.

Several citrus relatives such as *Poncirus trifoliata* and *Microcitrus* were considered as tolerant or resistant (Ramadugu et al., 2016; Godfrey et al., 2017), however, they are genetically distant from commercial citrus varieties which have originated mainly from three common ancestors, wild mandarin (*C. reticulata*), pummelo (*C. maxima*), and citron (*C. medica*) through a long domestication evolution (Wu et al., 2018). Rough lemon shares wild mandarin as a common ancestor with sweet orange. Our previous study showed that rough lemon is HLB tolerant compared with sweet orange (Fan et al., 2012). Once rough lemon trees are infected and symptomatic, they can be rejuvenated by the continued growth of new shoots with few or no foliar symptoms of the disease, and they repeat this cycle for many growing seasons; in contrast, sweet orange exhibits continuous growth inhibition and eventual dieback (Fan et al., 2012). By comparative transcriptional and anatomical analysis of rough lemon and sweet orange in response to *C*Las, phloem transport activity and the expression of defense-related genes are much greater in rough lemon than in sweet orange (Fan et al., 2012; Yu et al., 2017), suggesting the ability to maintain good phloem transport with extensive *C*Las titer is likely critical to good HLB tolerance. To further explore the contributions of lncRNA in response to HLB, we systematically identified lncRNAs from rough lemon and sweet orange at four different time points of a greenhouse experiment and characterized their genomic transcriptional and regulatory dynamics. We predicted their potential regulatory genes and functions and constructed a co-expression network. Our study provides valuable information and expands the knowledge of the role of citrus lncRNA in HLB disease expression.

## 2 Materials and methods

### 2.1 Plant materials

The plant inoculation was performed using the method as previously described (Fan et al., 2012). Briefly, two-year-old HLB-sensitive sweet orange (*C. sinensis* L Osb.) and HLB-tolerant rough lemon (*C. jambhiri* Lush.) plants were graft-inoculated with *C*Las positive bud wood collected from Carrizo citrange (*C. sinensis* × *P. trifoliata* L. Raf.) grown in a protected greenhouse, and mock-inoculated controls used bud wood from pathogen tested and healthy Carrizo trees. Each treatment had three biological replicates. All these plants were kept in a state-certified disease-free greenhouse (a United States Department of Agriculture Animal and Plant Health Inspection Service and Center for Disease Control-approved and secured greenhouse) at the University of Florida, Citrus Research and Education Center, Lake Alfred. Midribs of mature leaves were sampled from *C*Las-inoculated and mock-inoculated trees every two weeks after inoculation (WAI) at early stages (before ten weeks) and every one week at later stages; quantitative real-time PCR (qRT-PCR) was performed to test for the presence of *C*Las as previously described (Li et al., 2006). Plants were considered HLB positive when PCR cycle threshold (CT) values were below 30 (Yu et al., 2017). Positive plants were not detected until 17 WAI. The typical blotchy mottled HLB symptom was observed around 34 WAI in rough lemon and sweet orange. Based on the presence of positive samples and HLB symptoms, midribs of mature leaf from *C*Las-inoculated and mock-inoculated rough lemon and sweet orange trees at 0, 7, 17, and 34 WAI were collected. A total of 48 samples from four different time-points were used for RNA-seq in this study. The information about plant materials and the HLB test is shown in **Table S1**. Midribs from the mature leaves of rough lemon and sweet orange, which were under Huanglongbing (HLB) disease stress for more than ten years in the field, were also used for qPCR validation to explore whether the regulatory relationship between candidate lncRNAs and genes also existed in the plants from different growth condition.

### 2.2 Strand-specific RNA sequencing

Total RNA was extracted using TRIzol^®^ Reagent and purified using a RNeasy Mini Kit (Valencia, CA, United States) following the manufacturer’s protocol. Ribosomal RNA was removed from the total RNA using a Ribo-Zero rRNA removal kit (Epicenter-Illumina, Madison, WI, United States) following the manufacturer’s protocol. High-quality RNA was used to construct strand-specific RNA (ssRNA) libraries at the Interdisciplinary Center for Biotechnology Research (ICBR) Gene Expression Core, University of Florida (UF) described by Yu et al. (Yu et al., 2017). The prepared libraries were sequenced on an Illumina HiSeq 2000 (Illumina Inc., San Diego, CA, USA) producing paired-end 100 bp reads.

### 2.3 Transcript assembly and lncRNA identification

Low-quality reads and the adaptor sequences were removed using the fastp tool (Chen et al., 2018). After filtering, the clean reads were mapped to the *Citrus clementina* genome v1.0 (JGI) (Wu et al., 2014) using HISAT2 software (Kim et al., 2019). Next, StringTie was used to assemble transcripts of each sample, merge transcripts to get a consensus transcriptome assembly, and compute the abundance of these transcripts (Pertea et al., 2016). Subsequently, the newly assembled transcripts were compared with the *C. clementina* reference genome annotations using the GffCompare program (Pertea and Pertea, 2020). Transcripts overlapped with the known genes were discarded. The resulting transcripts with length ≥ 200 bp and fragments per kilobase of transcript per million fragments (FPKM) ≥ 1 in more than three samples were extracted, and then the tRNAs and rRNA were removed from the extracted transcripts using tRNAccan-SE (Lowe and Eddy, 1997) and RNAmmer (Lagesen et al., 2007), respectively. To reduce the noise of transcripts encoding proteins, TBtools software (Chen et al., 2020) was used to identify the open reading frames (ORFs) of these transcripts. Transcripts with significant ORFs were aligned to the Swiss-Prot, Nr, and Pfam databases, and the transcripts with E-value ≤ 10^-5^ were excluded. Finally, we further evaluated the coding ability of the remaining transcripts using the Coding Potential Calculator version2 (CPC2) (Kang et al., 2017). We searched the lncRNAs in the PLncDB V2.0 database (Jin et al., 2021) and CANTATAdb (Szcześniak et al., 2016) to identify novel lncRNAs. The information on lncRNAs is listed in **Table S2**. Based on the genomic coordinates of protein-coding genes, the lncRNAs were divided into five groups (Scheuermann and Boyer, 2013; Ransohoff et al., 2018): Intergenic lncRNAs (LINC), intronic lncRNA (INTRONIC), natural antisense transcripts (NAT), genic lncRNA (GENIC), and exonic lncRNA (EXON).

### 2.4 Prediction of lncRNAs targets, precursors, and eTMs

Mature miRNA and miRNA precursors were downloaded from the miRbase database (Release 22.1) (Kozomara et al., 2019). The psRNATarget (Dai et al., 2018) was used to predict lncRNAs acting as putative miRNA targets with the default settings. All 8742 identified lncRNAs were used to predict eTMs using the psMimic software with the default settings (Wu et al., 2013). The lncRNAs transcripts were aligned to the miRNA precursors sequences from the miRbase database to predict the precursors using BLASTN software based on the best hits with E-value < 1e-10 and query identify > 80%. The miRNA target of candidate genes was predicted using TAPIR (http://bioinformatics.psb.ugent.be/webtools/tapir/) online tool.

### 2.5 Differential expression analysis of lncRNAs and mRNAs

The expression level of lncRNAs and mRNAs were quantified based on the position of mapped reads using StringTie software (Pertea et al., 2016) and evaluated by FPKM. The sample biological replicates were examined using principal component analysis (PCA) and correlation analysis, and the differentially expressed (DE) analysis was performed using the DEseq2 package in R software (Love et al., 2014). DE mRNAs and lncRNAs were determined with false discovery rate (FDR) < 0.01 and fold change (FC) |log2FC| ≥ 1. Protein coding (PC) genes were annotated using Swiss-Prot (Boeckmann et al., 2003) and non-redundant (NR) database (Pruitt et al., 2005). Gene functional enrichment was analyzed using Metascape (Zhou et al., 2019) and MapMan (Thimm et al., 2004).

### 2.6 Orthologous identification and phylogenetic analysis of lncRNAs

The orthologous lncRNAs were identified based on reciprocal best blast hits (RHB) using the Basic Local Alignment Search Tool (BLAST) with query coverage > 80% and E-value < 1e-10 (Moreno-Hagelsieb and Latimer, 2008). The orthologous lncRNA sequences of *Arabidopsis thaliana*, *Oryza sativa*, and *Populus trichocarpa* were downloaded from the PLncDB V2.0 database (Jin et al., 2021). A total of 36 orthologues were identified. For phylogenetic analysis, sequences of 38 accessions (**Table S3**) were from previous publications except for *Citrus latipes* (Wu et al., 2014; Wu et al., 2018). Variant calling and filtering were according to the method previously described (Peng et al., 2020). Briefly, sequences were mapped to the *Citrus clementina* genome v1.0 (JGI) (Wu et al., 2014) *via* BWA-MEM (Li, 2013). Raw aligned reads were sorted and duplicate reads removed *via* samtools V1.7 (Li et al., 2009) and sambamba V0.6.7 (Tarasov et al., 2015), respectively. Variant calling and filtering were performed using The Genome Analysis Toolkit (GATK) v4.1.2 (McKenna et al., 2010) and VCFtools (Danecek et al., 2011), respectively. Finally, 1,658 bi-allelic variants derived from 36 conserved orthologous lncRNAs genomic loci were extracted and used to construct a maximum-likelihood (ML) phylogenetic tree (**Data S1**). The best substitution model general time-reversible (GTR) for the ML tree was inferred using Smart Model Selection (SMS) web server (Lefort et al., 2017).

### 2.7 Construction of co-expression network for lncRNAs and mRNAs

First, we excluded the transcripts that had similar expression patterns between mock-inoculated and *C*Las-inoculated plants to reduce the noise caused by gene spatiotemporal-specific expression using the ‘Mfuzz’ package in R software with the k-nearest neighbor method (Peterson, 2009). Co-expression analysis was performed using the weighted co-expression network analysis (WGCNA) package in R software with threshold power = 6, minimum module size = 3, and a branch merge cut height = 0.25 (Langfelder and Horvath, 2008). The co-expression network was plotted using Gephi software (Bastian et al., 2009). Proteins of *Arabidopsis thaliana* were used as model to infer the protein-protein interaction (PPI) of co-expressed genes in citrus, based on STRING database (Szklarczyk et al., 2015).

### 2.8 cDNA synthesis and qRT-PCR

Twelve genes with high amplification efficiency and primer specificity were selected to validate the RNA-seq data of rough lemon and sweet orange using qRT-PCR. In addition, we also validate *LNC28805*, *WRKY33*, and *SYP121* and their targeting *miRNA5021* in rough lemon and sweet orange, which were under Huanglongbing (HLB) disease stress for more than ten years in the field in Lake Alfred. The STEM-LOOP RT-qPCR method was carried out for *miRNA5021* based on the method of Kramer (2011) (Kramer, 2011). The qPCR method for mRNA and lncRNA was described by Yu et al. (2017). Briefly, the first strand cDNA was synthesized using an Affinityscript qPCR cDNA Synthesis Kit (Agilent Technologies, Santa Clara, CA, USA), and RT-qPCR was performed using SYBR Green qPCR Master Mix (Agilent Technologies) in a 20-μl volume. 18S rRNA gene was used for an internal reference according to previous studies (Yan et al., 2012; Wei et al., 2021). The primers are listed in **Table S4**.

## 3 Results

### 3.1 Identification and characterization of lncRNAs in citrus

**Figure 1:**
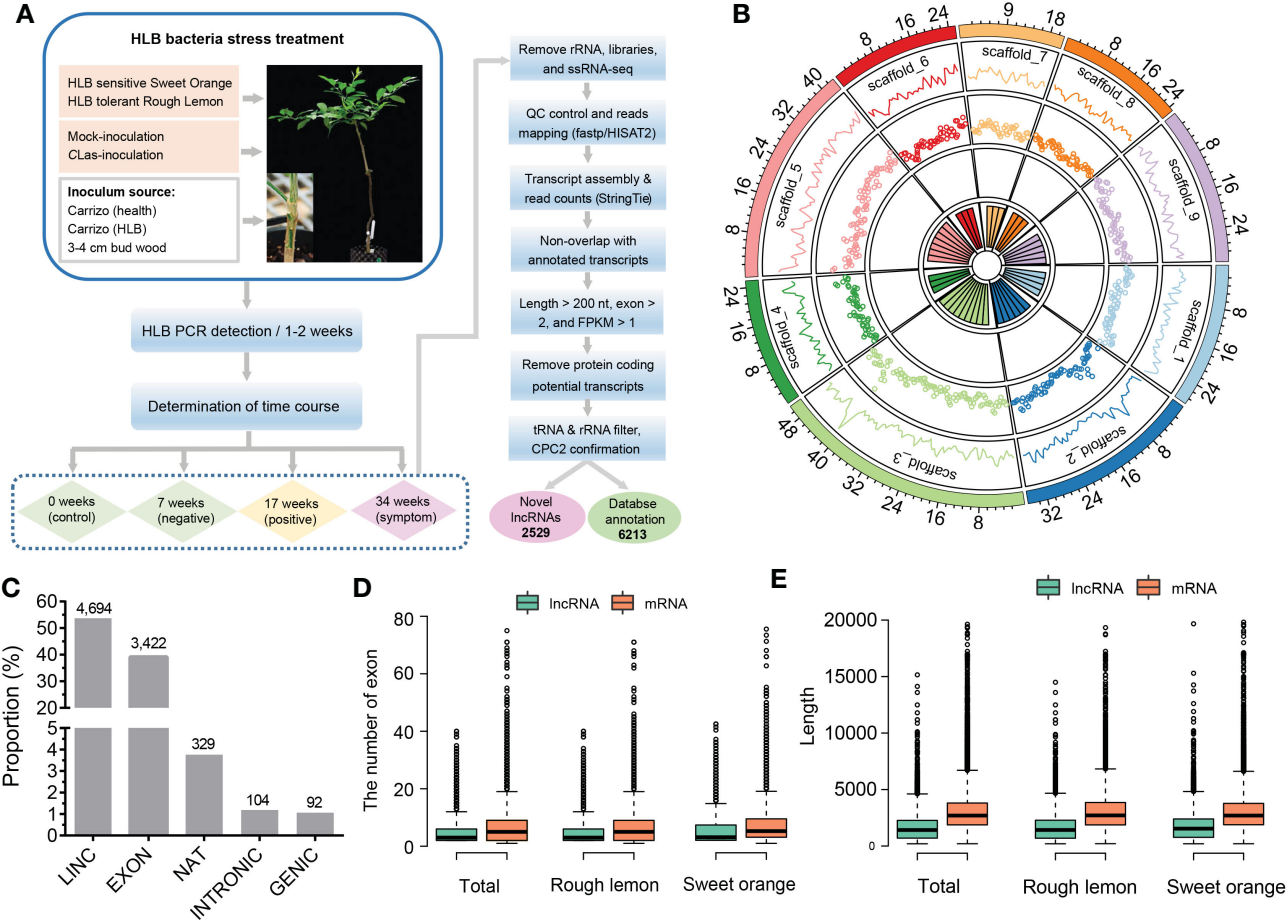
Identification and characterization of lncRNAs in HLB-tolerant rough lemon and HLB-sensitive sweet orange. **(A)** Pipeline of lncRNA identification. **(B)** Genomic distribution of lncRNAs on the *Citrus clementina* genome v1.0. From outer to inner rings indicates chromosome-level scaffolds, line plots, and dot plots of the lncRNA number distributed in each scaffold. **(C)** The numerical distribution of different types of lncRNAs. LINC, intergenic lncRNAs; INTRONIC, intronic lncRNA; NAT, natural antisense transcripts; genic lncRNA, GENIC; EXON, exonic lncRNAs. **(D)** Exon number and **(E)** length comparison of lncRNAs and mRNAs.

Approximately 2,523 million paired-end reads from 48 sample libraries were produced and mapped to the *C. clementina* v1.0 reference genome (**Table S5**). After a comprehensive pipeline of filtering, a total of 8,742 lncRNAs with FPKM > 1 in at least three samples were identified in both sweet orange and rough lemon, including 2,529 novel lncRNAs (**Figure 1A** and **Table S2**). Most of lncRNAs belong to LINC and EXON groups (**Figure 1C** and **Table S2**). The EXON group has a greater exon number and transcript length in comparison to other groups (**Figure S1A, B**).

To investigate the characteristics of citrus lncRNAs response to HLB, we analyzed the correlation coefficients between different samples using the expression profiles of lncRNAs. A relatively high correlation between biological replicates was observed (**Figure S2**). Analysis of lncRNAs distribution in the citrus genome showed that lncRNAs were widely expressed across all citrus chromosomes, and the highest number of expressed lncRNA was around the 40 Mb region on scaffold_3 (**Figure 1B**). We then investigated the transcript length and exon number distribution of lncRNAs and mRNAs. The exon number of lncRNA and mRNA had similar distribution patterns in rough lemon and sweet orange (**Figure S3**), while mRNA had a greater exon number and sequence length than lncRNA (**Figures 1D, E**; **Figure S3**). Meanwhile, we also investigated the difference in the overall gene expression levels of lncRNA and mRNA in four different periods. The results show that expression levels of mRNA were always higher than lncRNAs in both mock- and CLas-inoculated plants (**Figure S1C-D**).

### 3.2 Differential expression dynamics of lncRNAs and mRNA after CLas-inoculation

**Figure 2:**
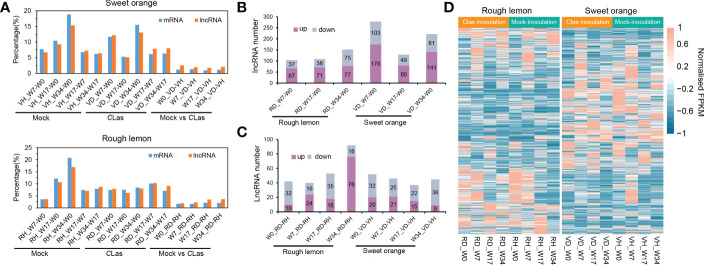
Global comparative analysis of lncRNAs and mRNA expressional dynamics. **(A)** Distribution of the differentially expressed (DE) mRNA and lncRNAs of 14 pairwise comparison groups in HLB-sensitive sweet orange and HLB-tolerant rough lemon. **(B)** Comparison of up- and down-regulated DE lncRNAs from W7-W0, W17-W0, and W34-W0 pairwise groups in *C*Las- inoculated rough lemon and sweet orange. **(C)** Comparison of up- and down-regulated DE lncRNAs between mock-inoculation and *C*Las-inoculation pairwise groups of rough lemon and sweet orange at the same stages. **(D)** Heatmap representing the expression patterns of DE lncRNAs in rough lemon and sweet orange. Data for lncRNAs expression were normalized to the Z-score.

The repeatability of samples was evaluated using PCA analysis. The VH1_W0 sample was eliminated due to poor replication (**Figure S4**). After that, twelve genes were selected to further validate the RNA-seq data, indicating good reliability of the data (**Figure S5**). Based on the analysis, we systematically compared the expression levels of lncRNAs and mRNAs in rough lemon and sweet orange plants at different time points of mock- and *C*Las-inoculated conditions. A total of 1,943 and 25,118 differentially expressed (DE) lncRNA and mRNAs were identified from 14 pairwise comparisons of rough lemon and sweet oranges (**Data S2-S3**). The percentage of DE lncRNAs and mRNAs was similar in both mock-inoculated sweet orange and rough lemon plants, but there was a prominent difference between them in comparisons of the *C*Las-inoculated groups (**Figure 2A**). A larger percentage of DE lncRNAs and mRNAs was found at week 7 and week 34 in *C*Las compared to groups of sweet orange, whereas there was no prominent difference in rough lemon (**Figure 2A**). These results indicate *C*Las altered the spatiotemporal expression pattern of plants and reflect the significant difference of genes in response to *C*las infection between sweet orange and rough lemon. We further investigated the number of up- and down-regulated DE lncRNA in sweet orange and rough lemon after *C*las-inoculation (**Figures 2B, C**). Comparing W0 and different time points after *C*Las inoculation, we found that the number of DE lncRNAs significantly increased at week 7 (W7) and week 34 (W34) in sweet orange (**Figure 2B**), and the largest number of DE lncRNA was at W7 (down/up, 103/176). However, the largest number of DE lncRNA was found at W34 in rough lemon, and only included 75 down-regulated and 77 up-regulated DE lncRNAs (**Figure 2B**). Comparing healthy plants with *C*Las infected plants at the same time point, we found that the number of upregulated DE lncRNAs significantly increased at W34 in rough lemon (down/up, 16/76), while it was the opposite in sweet orange at the same points (down/up, 36/9) (**Figure 2C**).

Heatmaps showed completely different expression patterns of lncRNAs between rough lemon and sweet orange species but a similar expression pattern in different time points of mock- and CLas-inoculated plants of the same species (**Figure 2D**), indicating lncRNAs have higher species specificity in response to *C*las infection. In addition, we further investigated the dynamic changes of specific DE lncRNAs and mRNAs in rough lemon and sweet orange (**Figure S6**). The number of specific DE lncRNAs in the pairwise groups of mock-inoculation vs. *C*Las-inoculation at the same time point was higher in rough lemon than in sweet orange at W17 and W34 groups (**Figure S6E**). However, in the pairwise groups of W0 -W7, W0-W17, and W0-W34 of *C*Las-inoculation, we found the number of specific DE lncRNAs of sweet orange was near two-fold higher than rough lemon (**Figure S6A**), and a mass of specific DE lncRNAs were found at week 7 and week 34 after *C*Las-inoculation, and most of them were upregulated in both rough lemon and sweet orange. However, a larger number of DE lncRNAs were downregulated in the same pairwise mock inoculation groups (**Figure S6B**). In addition, we also found the specific DE lncRNAs were overall upregulated in rough lemon or sweet orange in the other compared groups (**Figure S6C, D**).

### 3.3 Evolution and function of specific DE lncRNAs in citrus

**Figure 3:**
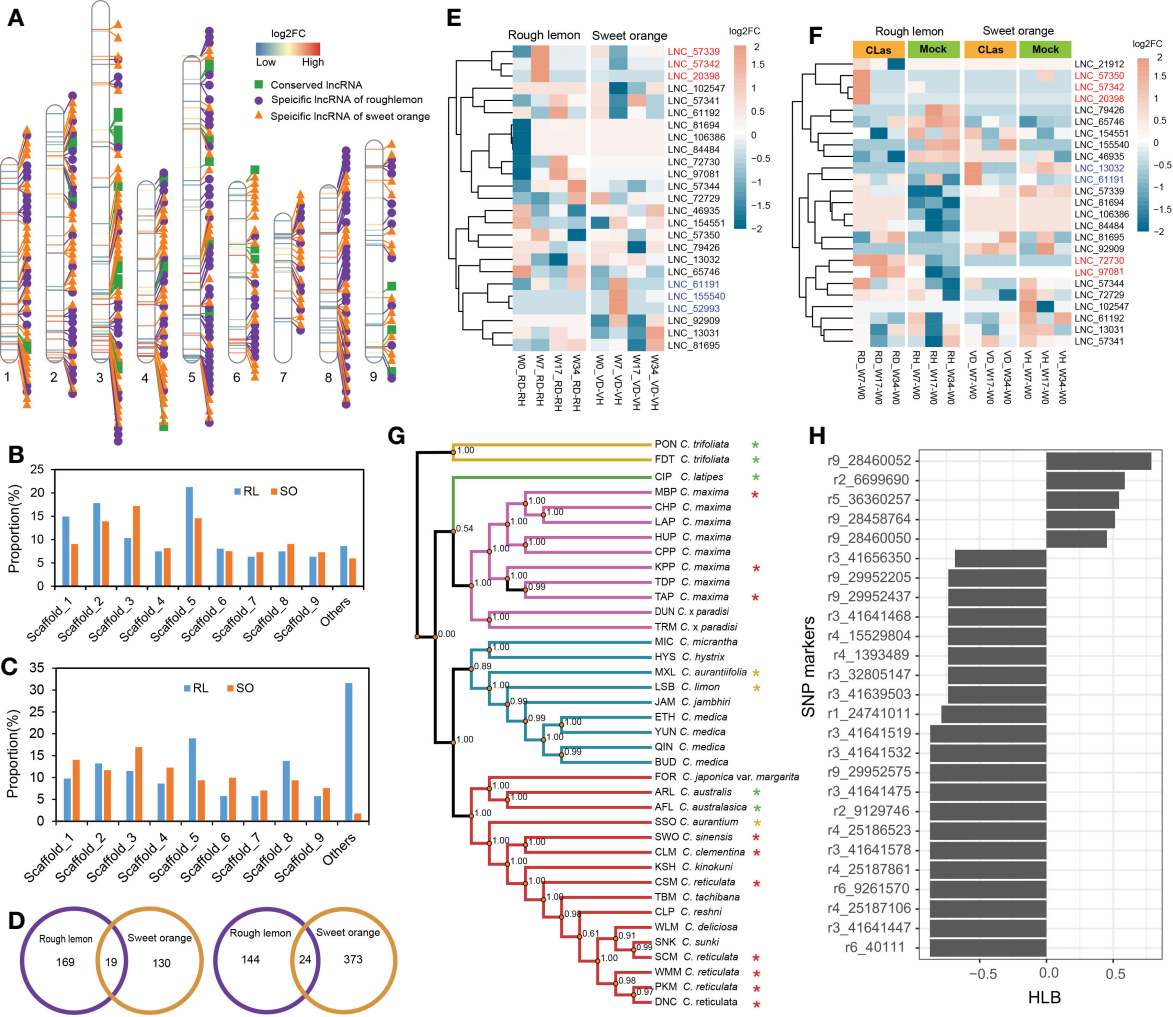
Identification and characterization of conserved lncRNAs in HLB-tolerant rough lemon and HLB-sensitive sweet orange. **(A)** Chromosome distribution of specific differentially expressed **(D, E)** lncRNAs and conserved DE lncRNAs. **(B)** Comparison of DE lncRNAs number of rough lemon and sweet orange distributed on the chromosome-level among W7-W0, W17-W0, and W34-W0 pairwise groups of *C*Las-inoculated plants, and **(C)** among comparison groups between mock- and *C*Las-inoculation at same time-point. RL, rough lemon; SO, sweet orange. **(D)** The number of DE lncRNAs specifically originated in the genome of rough lemon or sweet orange. DE lncRNAs of mock- and *C*Las-inoculated pairwise at same time-point (left); DE lncRNAs of W7-W0, W17-W0, and W34-W0 pairwise groups of *C*Las-inoculated plants (right). **(E, F)** Expression patterns of conserved lncRNAs across ten different pairwise comparison groups in rough lemon and sweet orange. Red and blue color font indicate lncRNAs significantly up-regulated at the early stage in rough lemon and in sweet orange, respectively. **(G)** A maximum likelihood (ML) phylogenetic tree of 38 citrus accessions. The tree was constructed based on the genomic variations derived from conserved lncRNAs. Asterisks indicate HLB tolerant levels (green, yellow, and red color indicate HLB tolerance, moderate tolerance, and sensitivity, respectively). The data of HLB evaluation were from Ramadugu et al. (2016) and Godfrey et al. (2017). **(H)** SNP markers derived from conserved lncRNAs significantly correlated with HLB response traits.

To further explore the characteristics of specific DE lncRNAs, we analyzed their genomic origins from rough lemon and sweet orange. The result shows that a total of 35 conserved lncRNAs, 166 specific lncRNAs in rough lemon, and 170 specific lncRNAs in sweet orange, were identified, and the distribution landscape of these specific DE lncRNAs was different between rough lemon and sweet orange on chromosome-level scaffolds (**Figure 3A**). Compared with the W0 time point (W7-W0, W17-W0, and W34-W0 pairwise groups of *C*Las-inoculated plants), most of DE lncRNAs were distributed in scaffold_1, 2, 3, 5, and their numbers exhibited significant differences between rough lemon and sweet orange (**Figure 3B**). The largest number was located on scaffold_5 and scaffold_3 in rough lemon and sweet orange, respectively (**Figure 3B**). In the pairwise groups between mock- and *C*Las-inoculated plants at the same time point, the largest percentage of DE lncRNAs (31.6%) was found in unanchored scaffolds in rough lemon, while the largest percentage was found on scaffold_3 in sweet orange (**Figure 3C**). We further investigated the number of common and unique genomic origin DE lncRNAs in rough lemon and sweet orange (**Figure 3D**). Only 43 specific DE lncRNAs derived from the same genomic loci of these two citrus species were identified, indicating that lncRNAs have high species-specific expression profiles in response to *C*Las.

Analysis of the evolutionary conservation of lncRNAs showed that thirty-one sequences of conserved lncRNAs located in seven different scaffolds were identified with e-value > 1×10^−10^ (**Figure 3A** and **Table S6**), and the expression levels of them represented specifically spatiotemporal different expression patterns between mock- and *C*Las-inoculated plants (**Figures 3E, F**). For instance, three lncRNAs (*LNC_57342*, *LNC_20398*, and *LNC_61191*) were significantly upregulated at W7 stage in rough lemon, while they were downregulated in sweet orange; the other three lncRNAs (*LNC_61191*, *LNC_155540*, and *LNC_52993*) exhibited reverse expression patterns in sweet orange (**Figure 3E**). Compared with W0, we found that seven lncRNAs exhibited higher differentially expressed levels in W7 and W17 time points after *C*Las inoculation in rough lemon and sweet orange. which might be involved in response to HLB in early phases after *C*Las inoculation. We further identified 1,658 bi-allelic variants derived from these homologous lncRNAs based on resequencing data of 38 citrus accessions, and a phylogenetic tree was constructed (**Table S3** and **Data S1**). The result showed that citrus species with close relatives were clustered together with higher support values (**Figure 3G**). Based on the HLB symptom evaluation of citrus relatives (Folimonova et al., 2009; Ramadugu et al., 2016; Godfrey et al., 2017), correlation analysis between bi-allelic variants and HLB symptom evaluation showed that 26 variants were significantly correlated with the HLB traits (**Figure 3H**). Most of the significant SNPs were located in 41.64-41.65 Mb region on scaffold_3 and 28.45-29.95 Mb region on scaffold_9. Six conserved DE lncRNAs with these correlated SNPs derived from four genomic loci (XLOC_007316, XLOC_030383, XLOC_072976, and XLOC_037329) were identified (**Table S7**).

In this study, we also predicted the miRNA targets, precursors, and eTMs of lncRNAs (**Data S4**). A total of 133 lncRNAs were identified as precursors for 33 miRNA families, 116 lncRNAs were identified to be targeted by 35 miRNA families, and 40 lncRNAs were predicted as eTMs for 14 miRNA families. Notably, 16 lncRNAs predicted as miRNA targets were simultaneously acting as miRNA precursors or eTMs, and nine lncRNAs were differentially expressed in sweet orange (**Table S8**), suggesting that these DE lncRNAs may be involved in response to HLB by interacting with miRNAs in sweet orange.

### 3.4 Identification of lncRNA-mRNA co-expression modules related to HLB response

**Figure 4:**
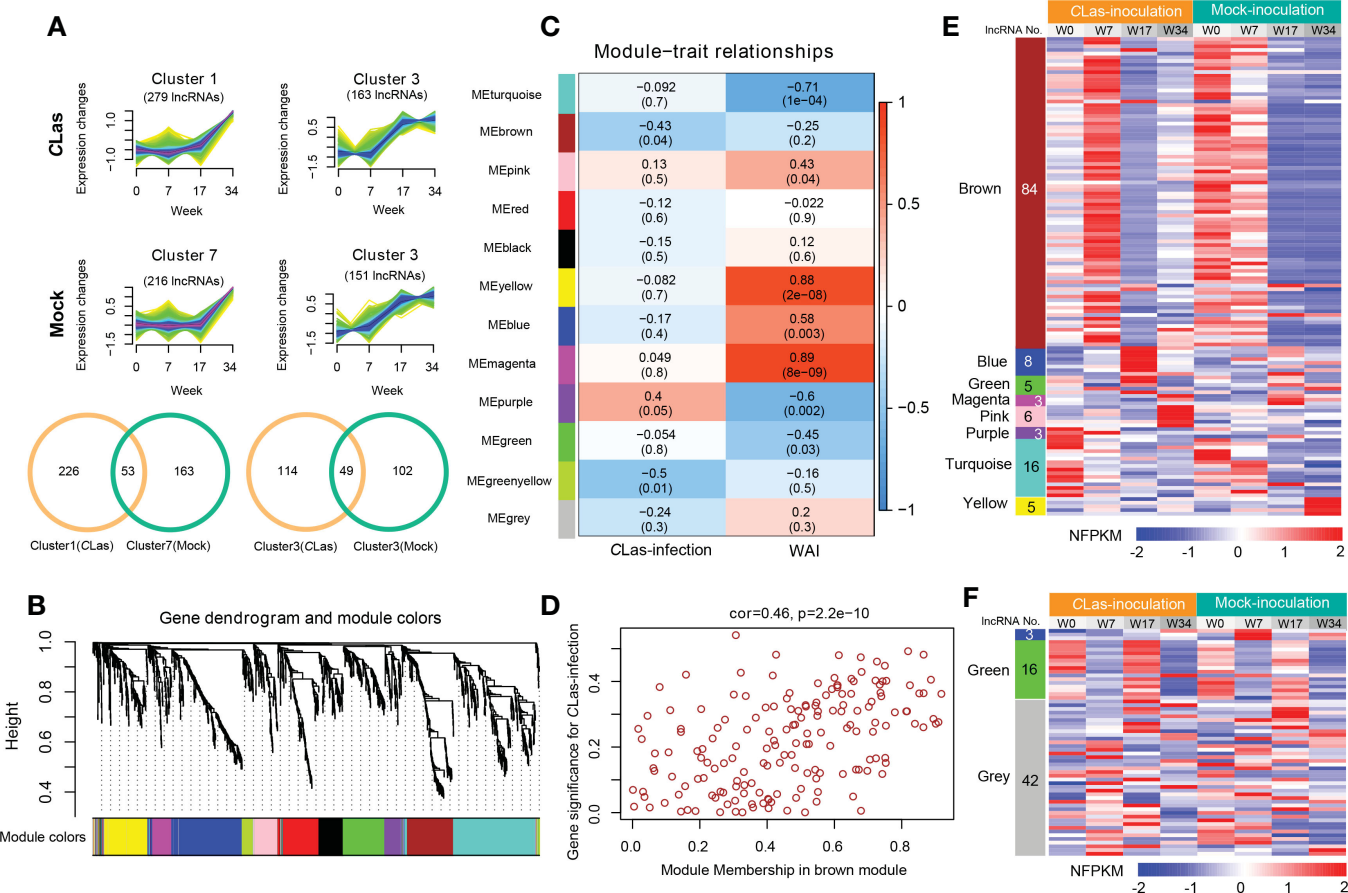
Hierarchical clustering and lncRNA-mRNA co-expression modules in rough lemon. **(A)** Similar expression tendency of lncRNAs in *C*Las-inoculation and mock-inoculation plants. Venn diagrams showing the specifically expressed lncRNAs. **(B)** Hierarchical cluster tree showing lncRNAs and mRNAs co-expression modules identified by Weighted Gene Co-expression Network Analysis (WGCNA). Twelve different modules were constructed and labeled by different colors. **(C)** Module-*C*Las-infection and module-WAI (weeks after inoculation) relationships. Each row corresponds to a module; Left column corresponds to inoculation approach and right column corresponds to the time point of after inoculation. Each cell is color-coded by correlation coefficient and contains corresponding *P-value*. **(D)** A scatterplot showing the relationship between gene significance for inoculation and module membership in brown module of rough lemon. **(E, F)** Heatmap showing the normalized FPKM (NFPKM) of lncRNAs in each significant module in **(E)** rough lemon and **(F)** sweet orange. FPKM were normalized to the Z-score.

To identify the DE lncRNAs and mRNAs potential response to HLB and to reduce the noise caused by gene spatiotemporal-specific expression, we first excluded the DE lncRNAs or mRNAs that had similar expression patterns in different time points of mock- and *C*Las-inoculation plants by using the k-nearest neighbor method (**Figure 4A** and **Figure S7, 8**). For instance, 53 and 49 similar expression pattern lncRNAs were excluded in the comparison of cluster1 (*C*Las) vs. cluster7 (Mock) and cluster3 (*C*Las) vs. cluster3 (Mock) in rough lemon (**Figure 4A**), respectively. Finally, a total of 2133 (including 246 lncRNAs and 1887 mRNAs) and 2863 (including 295 lncRNAs and 2568 mRNAs) transcripts from rough lemon and sweet orange, respectively, were potentially responsible for HLB response and were used for co-expression analysis. This analysis resulted in 12 and 7 distinct modules, which are clusters of highly interconnected genes (Langfelder and Horvath, 2008) in rough lemon (**Figure 4B**) and sweet orange (**Figure S9A**), respectively. Module eigengene is considered a representative gene expression profile in a module and correlated with the corresponding tissue type or trait (Langfelder and Horvath, 2008). We found that 3 out of 12 co-expression modules are comprised of genes that are significantly correlated with Mock- or *C*Las-inoculation, and 7 out of 12 modules are significantly correlated with time points (0, 7, 17, 34 WAI) (*P* ≤ 0.05; **Figure 4C**) in rough lemon. However, no significant modules were correlated with *C*Las inoculation in sweet orange except for three significant modules correlated with the WAI (**Figure S9B**).

Modules with high trait significance may represent potential pathways associated with the trait, and genes with high module membership in modules significantly correlated with traits are hub candidate genes (Langfelder and Horvath, 2008). In this study, scatterplots showed that genes with high module membership in 8 modules in rough lemon (**Figure 4D** and **Figure S10**) and three modules in sweet orange (**Figure S9C**) were identified as potential hub genes with high significance (*P* < 0.01), including a total of 130 lncRNAs and 61 lncRNAs in rough lemon and sweet orange, respectively (**Table S9**). Among these lncRNAs, 23 lncRNAs were predicted to function as miRNA targets, miRNA precursors, or eTM (**Table S10**).

A heatmap showing the relative normalized FPKM (NFPKM) of lncRNAs revealed that most of the co-expression lncRNAs were from brown module correlated with *C*Las-inoculation and significantly upregulated at 7 WAI in rough lemon, and lncRNAs in different modules exhibited highly specific expression (**Figure 4E**). However, few co-expression lncRNAs showed spatiotemporal-specific expression in sweet orange (**Figure 4F**). Heatmap of co-expressed mRNAs also showed prominently different expression patterns between rough lemon and sweet orange (**Figure S11**). Genes in the pink and blue modules were specifically upregulated at 17 WAI and 34 WAI after CLas-inoculation in rough lemon, respectively (**Figure S11A**), while genes in the blue module of sweet orange were significantly downregulated at 7 WAI and 34 WAI (**Figure S11B**). These specific DE lncRNAs and mRNAs might specifically contribute to the response to *C*Las infection.

### 3.5 Functional annotation and enrichment of genes in significant modules

**Figure 5:**
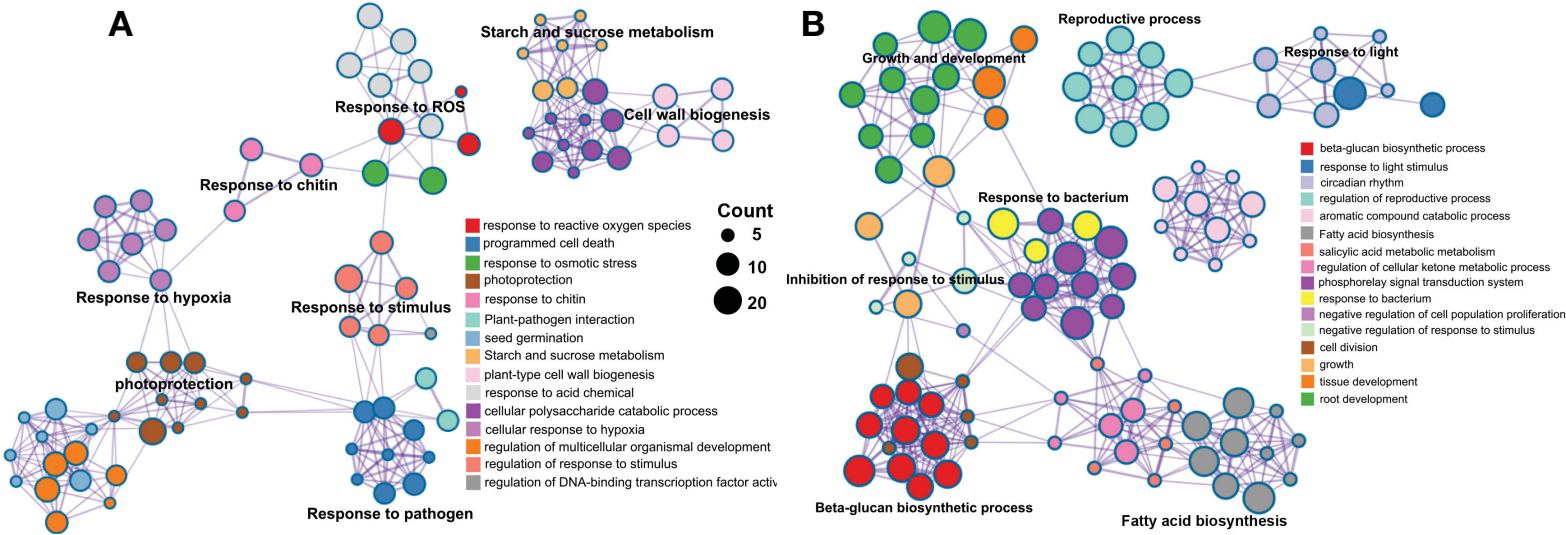
Pathway and process enrichment analysis of genes in the significant WGCNA modules in **(A)** rough lemon and **(B)** sweet orange. Network of enriched terms colored by cluster ID, where nodes that share the same cluster ID are typically close to each other.

A total of 1146 and 879 were included in the eight significant modules in rough lemon and three significant modules in sweet orange, respectively. GO enrichment indicated that stress response-related biological process terms (such as response to reactive oxygen species, plant-pathogen interaction, and response to stimulus processes) were significantly over-represented in rough lemon (**Figure 5A** and **Figure S12A**). However, the development and growth of biological processes, negatively regulated cell proliferation, and response to stimulus terms were significantly over-represented in sweet orange (**Figure 5B** and **Figure S12B**). MapMan functional categories related to biotic stress show that redox state, glutathione-S-transferase, and secondary metabolites were significantly enriched in rough lemon (**Figure S12C**). Notably, genes enriched in the Glutathione-S-transferase category were mainly involved in pathogenic effector-triggered immunity, systemic acquired resistance, and WRKY33-dependent plant immunity in rough lemon, and most of these genes were significantly upregulated (**Data S5** and **Figure S12C**). In sweet orange, most genes were mainly enriched in the redox state and secondary metabolites categories related to cell wall organization, but few genes were enriched in the Glutathione-S-transferase category related to pathogen response (**Data S5** and **Figure S12D**). This result indicates pathogenic response genes in the Glutathione-S-transferase category play an important role in HLB tolerance of rough lemon. In addition, we also found that the functional roles of genes in the redox state and secondary metabolite categories were quite different between rough lemon and sweet orange. Compared with rough lemon, many cell wall related genes were enriched in sweet orange, including genes related to arabinogalactan, callose, and lipid biosynthesis (**Data S5**). According to the previous study, callose deposition can play a role as a defensive fortification in response to *C*Las bacteria in citrus (Achor et al., 2010). Based on the results of gene function enrichment, we suggest that the mechanisms of HLB response are quite different between rough lemon and sweet orange.

### 3.6 Co-expression regulatory network of lncRNA-mRNA in response to *C*Las infection

Based on the results of GO and MapMan functional enrichment, co-expression genes associated with pathogen stimuli response in rough lemon and genes associated with callose synthase in sweet orange were of particular interest (**Table S11**). Multiple genes (such as NLR, RIN4, and CBP60/SARD) were suggested to play an important role in plant innate immunity (Abramovitch et al., 2006; da Graça et al., 2016). Genes showing the most connections with those immunities might be mutually involved in the pathway of pathogen response.

Therefore, we further identified genes first connected to stimuli response and callose synthase related genes and constructed their potential co-expression regulatory network. A total of 205 and 73 first neighboring genes with weight > 0.2 were identified in rough lemon and sweet orange, respectively (**Figure S13A, B** and **Data S6**). Multiple defense-related genes were identified in the network, such as *BOTRYTIS-INDUCED KINASE 1* (*BIK1*)/MSTRG.37943.1 and *NECROTIC SPOTTED LESIONS 1* (*NLS1*)/MSTRG.17571.7 in rough lemon; peroxidase superfamily protein (*PRX52*)/*MSTRG.57748.1* and subtilisin-like protease (*SBT1.5*)/*MSTRG.58400.1* in sweet orange. GO enrichment showed that the neighboring genes in rough lemon and sweet orange were significantly enriched in plant-type hypersensitive response and cutin biosynthetic process, respectively (**Figure S13C-D**). In addition, seven pathogen-related genes derived from MapMan pathogen stimuli response category showed a strong association with each other (**Figure S13A**). Among these seven genes, effector-triggered immunity related *RPM1-interacting factor 4* (*RIN4*)/*MSTRG.29058.1* and WRKY33-dependent plant immunity related SIGMA FACTOR BINGD PROTEIN (SIB)/Ciclev10002803m.v1.0 with the highest edge weight are suggested to be two key hub genes in the co-expression network.

**Figure 6:**
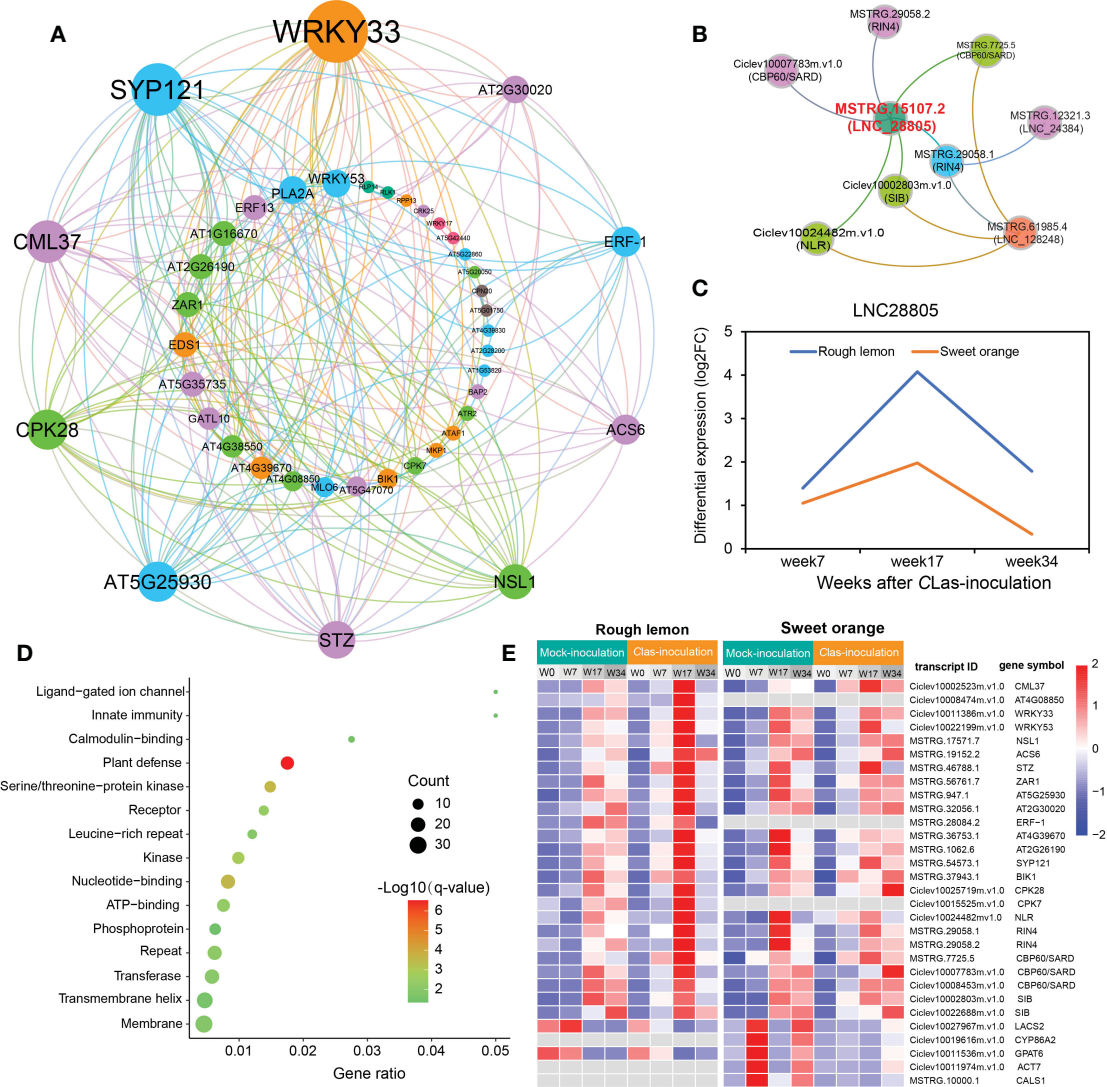
Construction of pathogen-related protein-protein interaction (PPI) network and lncRNA-mRNA co-expression network in response to *C*Las infection. **(A)** PPI network of co-expressed genes based on the prediction of the STRING database with edge confidence > 0.4. *A thaliana* was used as model to infer the PPI network of the co-expressed genes in citrus species. The circle size indicates the edge degree of the adjacent genes in the PPI network; nodes and edges were colored by modularity class based on Gephi software analysis. **(B)** lncRNAs co-expressed with pathogen stimuli response genes. Red font indicates the most significant hub lncRNA. **(C)** Differential expression of *LNC28805* across three different stages after *C*Las inoculation. FC indicates fold-change. **(D)** Functional enrichment of 145 co-expressed differentially expressed transcripts first connected to *LNC28805*. **(E)** Heatmap showing the expression patterns of co-expressed transcripts related to pathogen response in rough lemon and sweet orange. FPKM were normalized to the Z-score.

To know more about the protein-protein interaction (PPI) relationships between these connected genes, proteins of *A. thaliana* were used as model to infer the potential interaction network (Szklarczyk et al., 2015). A total of 43 and 13 PPI genes (edge confidence > 0.4) were identified in rough lemon and sweet oranges, respectively (**Figure S14** and **Data S7**). Most of them in the PPI network were related to stress response (**Figure 6A**; **Figure S14**). For instance, WRKY33 (Ciclev10011386m.v1.0) is important for plant pathogen immunity and interacted with multiple disease related proteins in rough lemon. ACS6 is involved in bacterial flagellin-induced ethylene production (Gravino et al., 2015), SYP121 is involved in ABA signaling (Eisenach et al., 2012), NECROTIC SPOTTED LESIONS (NSL1) negatively regulated cell death programs, and salicylic acid-related defense responses; *nsl1* mutants exhibited higher levels of salicylic acid (SA) and callose deposition (Noutoshi et al., 2006). As to the first neighboring genes of callose synthase in sweet orange, LONG-CHAIN ACYL-COA SYNTHETASE 2 (LACS2) is required for the accumulation of cuticular wax to enhance plant stress resistance (**Figure S14B**), which strongly interacts with CYP86A2 and is involved in the processes of cuticle development and repression of bacterial type III gene (Xiao et al., 2004). Notably, WRKY33 and its interacted SIB (Ciclev10002803m.v1.0) had the highest confidence in the PPI and co-expression networks (**Figure 6A** and **Figure S13A**), implying an extent regulatory relationship between them in response to *C*Las infection. Based on the PPI network, genes with high confidence (edge confidence > 0.7) were identified, and a set of them related to pathogenic response, such as effector receptor, systemic acquired resistance protein, callose synthase, and calcium-dependent protein et al., are identified as key genes in response to *C*Las infection (**Table 1**). The analysis of gene expression showed that pathogen response genes were distinctly upregulated in rough lemon at 17 weeks after *C*Las inoculation, while callose synthase related *CALS1* was not expressed in rough lemon (**Figure 6E**). Interestingly, effector-triggered immunity related leucine-rich repeat receptor-like protein kinase family protein (*AT4G08850*), pathogen-associated molecular pattern (PAMP)-triggered immunity (PTI) signaling related *CPK7*, and ethylene signaling response gene *ERF-1* were not expressed in sweet orange, which partially explained greater HLB tolerance.

**Table 1:** Function and annotation of key genes related to disease response in the co-expression networks.

Transcript ID	Arabidopsis homolog	Description	Function	miRNA target
Ciclev10002523m.v1.0	CML37	Calcium-binding protein CML37	Potential calcium sensor	
Ciclev10008474m.v1.0	AT4G08850	Leucine-rich repeat receptor-like protein kinase family protein	Plant immunity and defense response	
Ciclev10011386m.v1.0	WRKY33	Probable WRKY transcription factor 33	Mediating responses to the bacterial pathogen and the necrotrophic pathogen.	ath-miR5021
Ciclev10022199m.v1.0	WRKY53	Probable WRKY transcription factor 53	Regulate the early events of leaf senescence	
MSTRG.17571.7	NSL1	MAC/Perforin domain-containing protein	Negatively regulating salicylic acid-related defense responses and cell death programs	–
MSTRG.19152.2	ACS6	1-aminocyclopropane-1-carboxylic acid (acc) synthase 6	Involved in bacterial flagellin-induced ethylene production	ath-miR5021
MSTRG.46788.1	STZ	Related to Cys2/His2-type zinc-finger proteins	Acts as a transcriptional repressor and is responsive to chitin oligomers	–
MSTRG.56761.7	ZAR1	Disease resistance RPP13-like protein 4	CC-NB-LRR receptor-like protein required for recognition of the Pseudomonas syringae type III effector HopZ1a	–
MSTRG.947.1	AT5G25930	Protein kinase family protein with leucine-rich repeat domain	Involved in protein amino acid phosphorylation; a crucial component of early immune responses	–
MSTRG.32056.1	AT2G30020	Protein phosphatase 2C family protein	Negatively regulates defense response; inactivates MPK4 and MPK6	–
MSTRG.28084.2	ERF-1	Ethylene responsive element binding factor 1	Ethylene signaling response	–
MSTRG.36753.1	AT4G39670	Glycolipid transfer protein (GLTP) family protein	Involved in glycolipid transport	ath-miR5021
MSTRG.1062.6	AT2G26190	IQ domain-containing protein IQM4	Involved in biotic and abiotic stress responses	–
MSTRG.54573.1	SYP121	Syntaxin of plants 121	A component of a complex of SNARE proteins that plays a role in ABA signaling and against fungal invaders	ath-miR5021
MSTRG.37943.1	BIK1	Serine/threonine-protein kinase BIK1	Required to activate the resistance responses to necrotrophic pathogens	
Ciclev10025719m.v1.0	CPK28	Calcium-dependent protein kinase 28	Involved in pathogen-associated molecular pattern (PAMP)-triggered immunity (PTI) signaling	–
Ciclev10015525m.v1.0	CPK7	Calcium-dependent protein kinase 7	Involved in pathogen-associated molecular pattern (PAMP)-triggered immunity (PTI) signaling	–
Ciclev10024482m.v1.0	NLR	Disease resistance protein (TIR-NBS-LRR class) family	Effector receptor; involved in signal transduction, defense response, apoptosis, innate immune response;	
MSTRG.29058.1 MSTRG.29058.2	RIN4	RPM1-interacting factor	The plant immune regulator; required for activation of RPM1-dependent inhibition of bacterial growth.	
MSTRG.7725.5 Ciclev10007783m.v1.0 Ciclev10008453m.v1.0	CBP60/SARD	calmodulin binding protein 60 (CBP60) family transcription factors	Systemic acquired resistance (SAR) positively regulate immunity	
Ciclev10002803m.v1.0 Ciclev10022688m.v1.0	SIB	Sigma factor binding protein (SIB)	Stimulate the DNA binding activity of WRKY33	
Ciclev10027967m.v1.0	LACS2	Long chain acyl-CoA synthetase 2	Activation of long-chain fatty acids for both synthesis of cellular lipids, and degradation *via* beta-oxidation	–
Ciclev10019616m.v1.0	CYP86A2	Cytochrome P450, family 86, subfamily A, polypeptide 2	Involved in the biosynthesis of hydroxylated fatty acids r, cuticle development and repression of bacterial type III gene expression	–
Ciclev10011536m.v1.0	GPAT6	Glycerol-3-phosphate 2-O-acyltransferase 6	Esterifies acyl-group from acyl-ACP to the sn-2 position of glycerol-3-phosphate, a step in cutin biosynthesis	–
Ciclev10011974m.v1.0	ACT7	Actin-7	Involved in the regulation of hormone-induced plant cell proliferation and callus formation	–
MSTRG.10000.1	CALS1	Callose synthase 1	Involved in callose synthesis at the forming cell plate during cytokinesis	–

Next, we further investigated the co-expression relationship between pathogen stimuli response genes and lncRNAs. We found three lncRNAs (*LNC_28805*, *LNC_24384*, and *LNC_128248*) that were co-expressed with pathogen response genes in rough lemon (**Figure 6B**). In this network, *LNC_28805* was a hub lncRNA first connecting to all five pathogen stimuli response genes, suggesting it might be a key regulator involved in response to *C*Las. LncRNAs could regulate gene transcription levels and cellular processes by acting as miRNA precursors or miRNA mimics (Chekanova, 2015). Among the three lncRNAs, *LNC28805* was solely found to act as *miR167* and *miR5021* targets in both rough lemon and sweet orange and exhibited similar expression patterns after *C*Las inoculation (**Table S10** and **Figure 6C**). *LNC28805* was significantly upregulated at 17 WAI in rough lemon. To realize whether other pathogenic response genes could be co-expressed with *LNC28805*, we further identified the first neighboring genes of *LNC28805* with edge weight > 0.2 from rough lemon and sweet orange. A total of 145 differentially expressed transcripts first connect to *LNC28805* in rough lemon (**Data S8**). However, none of the genes co-expressed with *LNC28805* were found in the co-expression network of sweet orange. Functional enrichment analysis of the 145 transcripts showed that plant defense-related genes were the most significantly enriched (**Figure 6D**).

### 3.7 A hypothetical regulatory pathway of *LNC28805* involved in HLB regulation

**Figure 7:**
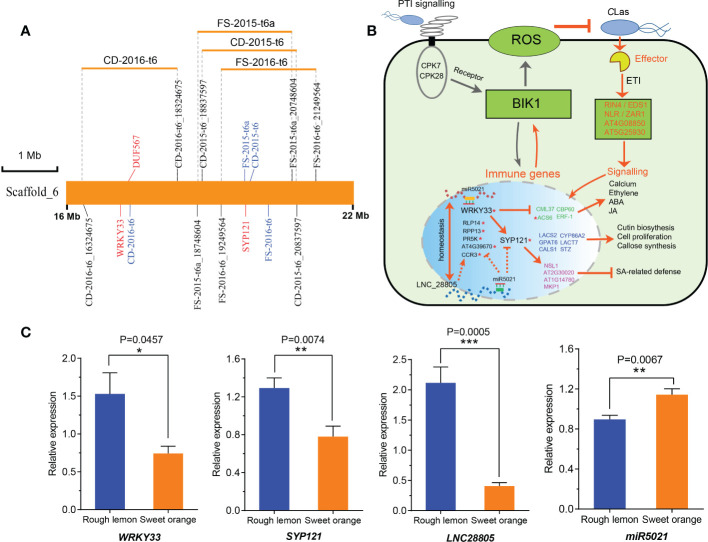
A hypothetical regulatory pathway of *LNC28805* involved in citrus HLB regulation. **(A)** Most significant hub genes targeted by *miRNA5021* located in an overlapping QTL identified by Huang et al., 2018. Word colored by blue indicates the QTL peak marker; CD, canopy damage; FS, foliar symptom. **(B)** A hypothetical regulatory model depicting the *LNC28805* and co-expression genes related to disease response based on protein-protein interaction (PPI) networks (shown in **Figure 6A** and **Figure S14**) and reported studies. This network indicated that *WRKY33* and defense related genes targeted by *miR5021* putatively regulate cross-talk between jasmonate-, abscisic acid (ABA)-, and salicylic acid (SA)-regulated pathways (Zheng et al., 2006; Birkenbihl et al., 2012; Liu et al., 2015). *LNC28805* might importantly compete with endogenous *miR5021* to maintain the homeostasis of these immune gene expression levels. Red asterisks indicate *miR5021* targets. **(C)** Relative expression of *miRNA5021* and its targets in rough lemon and sweet orange under Huanglongbing (HLB) stress more than ten years in the field (shown in **Figure S15**). Asterisks indicate significant differences (Students’ t-test, *p < 0.05, **p < 0.01, ***p < 0.001).

*LNC28805* was not predicted to act as an eTM or miRNA precursor, but it did appear to act as a target for *miR167* and *miR5021*, thus suggesting a role in the regulation of HLB response by interacting with miRNAs. To validate this point, we analyzed the *miR502*1 and *miR167* potential targets among the co-expression genes of *LNC28805*, as well as pathogen stimuli response genes and callose synthase genes. A total of 25 transcripts were identified to be targeted by *miR5021* in rough lemon and sweet orange, respectively (**Table S12** and **Data S9**). However, none of the transcripts were identified to be targeted by *miR167*. Most of these genes were involved in disease regulation, such as *WRKY33*, *SYP121*, and *NB-ARC* domain-containing disease resistance protein (**Table S12**). Interestingly, we found eight genes targeted by *miR5021* were found with the QTLs identified in our previous study (Huang et al., 2018) (**Table S13**). Intriguingly, QTLs on scaffold_6 were simultaneously detected for foliar symptoms (FS) and canopy damage (CD) in two different years, and there two key hub genes *(WRKY33* and *SYP121*) in the PPI network, strongly linked to the QTL peak markers, were found (**Figure 7A**). This result further supports a putatively important regulatory relationship between *LNC28805* and these disease response genes. It is tempting to speculate that *LNC28805* might act as competing endogenous *miR5021* to regulate HLB response genes by attenuating their cleavage or translated inhibition caused by *miR5021*. The mechanism of miRNA-LncRNA interactions regulating host immunity-related genes has been reported in tomato (Jiang et al., 2020). Overall, a hypothetical model of *LNC28805* and its potential role in regulatory processes was proposed (**Figure 7B**). This model showed that WRKY33 might regulate cross-talk between jasmonate-, abscisic acid (ABA)-, and salicylic acid (SA)-regulated disease response pathways (Zheng et al., 2006; Birkenbihl et al., 2012; Liu et al., 2015).

To explore if the regulatory relationship also exists in the plants under HLB stress for a long term in the field, we further tested the expression levels of *miR5021* and its targets (*WRKY33*, *SYP121*, and *LNC28805*) using midribs of rough lemon and sweet orange under HLB stress more than ten years in the field (**Figure S15**). We found *LNC28805* was significantly upregulated, and *miR5021* was significantly downregulated in rough lemon, which supports the hypothesis that *LNC28805* is involved in regulating the expression level of pathogenic response genes by competing for endogenous *miR5021*.

## 4 Discussion

It has been known that lncRNAs act as versatile regulators involved in diverse biological processes in plants. In citrus, thousands of lncRNAs from different species have been identified in flowers, leaves, seeds, and fruits based on RNA-seq transcriptome data (Xu et al., 2018). However, lncRNAs have not been systematically identified in response to citrus HLB, and little is known about their biological function during the response to the disease. Here, we present a comprehensive picture of lncRNAs and mRNAs transcription patterns in different time points after *C*Las-inoculation in HLB-tolerant rough lemon and HLB-sensitive sweet orange. A set of DE lncRNAs exhibited stage-specific and species-specific expression, implying specific roles for lncRNAs in response to infection of citrus with *C*Las, leading to HLB.

### 4.1 Identification, conservation, and phylogenetic relationship of lncRNAs

In this study, we have identified 8742 high confident lncRNAs from mock- and *C*Las-inoculated rough lemon and sweet orange. There are more than 50% lncRNAs from intergenic regions, which is consistent with a study of lncRNAs and flowering in trifoliate orange (Wang et al., 2017). We also identified 2529 novel lncRNAs by aligning lncRNA sequences to the annotated lncRNA database (Szcześniak et al., 2016; Jin et al., 2021). These lncRNAs might specifically respond to HLB disease and need more investigation. Therefore, our study has expanded the information on disease-related lncRNAs in citrus. Compared to protein-coding genes, lncRNAs identified from rough lemon and sweet orange shared most of the common features of lncRNAs reported in other citrus species and model plants (Di et al., 2014; Yuan et al., 2018; Ke et al., 2019), such as low conservation, fewer exons, shorter transcript length, and lower expression levels. Similar features in different species might be explained by the high rates of origin sequence evolution (Mercer et al., 2009). These results are consistent with previous studies in other species (Ke et al., 2019; Tang et al., 2021), also supporting the reliability of lncRNAs identified in this study. Analysis of the primary sequence evolutionary conservation by comparing with *A. thaliana*, *O. sativa*, and *P. trichocarpa* lncRNAs showed that only 33 lncRNAs derived from 22 genomic loci were identified. Compared with *Arabidopsis* and rice, a relatively high proportion of conserved lncRNAs was found in *P. trichocarpa*, indicating high sequence similarity in a more closely related taxon. The phylogenetic tree of 38 citrus relatives also displayed high conservation of these lncRNAs genomic sequence at the intra-species level (**Figure 3G**), suggesting that these conserved lncRNAs might share a common ancestor and ancient evolutionary origin at genomic levels (Ulitsky, 2016). Similar results were also observed in animals and other plants (Pang et al., 2006; Di et al., 2014). Intriguingly, correlation analysis between SNPs derived from the conserved lncRNAs indicated 26 SNPs were significantly associated with HLB tolerance, and six conserved lncRNAs contained these SNPs were significantly upregulated at W7 time point after *C*Las-inoculation (**Figure 3E** and **Table S7**). Some studies reported that conserved lncRNAs might play an important role in conserved functions, such as interacting with RNA-binding proteins by conserving secondary structures (Wang et al., 2014; Ulitsky, 2016). Although experimental evidence about the biogenesis and functions of conserved lncRNAs is limited, most current studies suggest specific secondary structures of conserved lncRNAs might be important for a conserved function (Johnsson et al., 2014; Ulitsky, 2016). More extensive experiments are needed to validate whether these conserved lncRNAs have important contributions to HLB response at an early stage in our future studies.

### 4.2 Tissue-specific and species-specific expression pattern of lncRNAs

It is difficult to deduce and validate the lncRNA function due to its low conservation and non-coding property. The tissue-specific expression may help to understand the potential functions underlying lncRNAs. In the current study, lncRNAs exhibited more prominent species-specific expression patterns compared with spatiotemporal-specific expression patterns (**Figure 2D**). Pairwise comparisons between mock- or *C*Las-inoculated tissues or different time points revealed that the number of DE lncRNAs and mRNAs distinctly increased at week 7 and decreased at week 17 after *C*Las inoculation in sweet orange (**Figure 2A**). However, it did not change significantly in rough lemon. The greater numbers of DE lncRNAs and mRNAs in sweet orange may imply greater sensitivity and activity in response to external stimuli at transcriptional regulation levels. Changes in the environment and rhythmic plant growth also can affect plant gene expression levels (López-Maury et al., 2008; Covington et al., 2008). The pairwise comparison of lncRNAs between mock- and *C*Las-inoculated plants was further performed to reduce the environmental noise, presenting obvious differences of lncRNA number at 34 WAI between rough lemon and sweet orange (79 upregulated lncRNAs in rough lemon and nine upregulated lncRNAs in sweet orange). Moreover, most of these lncRNAs presented high species-specificity. These results indicated that species-specific DE lncRNA might play an essential role in the regulation of HLB tolerance in rough lemon. High tissue- and species-specific expression of lncRNAs, such as *LNC_57342* and *LNC_61191* specifically expressed in rough lemon and sweet orange at W7 time point, respectively, also make them potentially useful as biomarkers for HLB detection or screening HLB tolerant species in early stages. Based on the aforementioned results, we suggest these specific DE lncRNAs might contribute to citrus HLB tolerance or sensitivity and imply important dynamics of lncRNAs response to HLB in citrus.

### 4.3 Putative interaction between DE lncRNAs and miRNAs

The lncRNA-miRNA interaction is a vital regulatory mechanism of lncRNAs. Several studies have shown that lncRNAs can serve as miRNA and siRNA precursors to assist in target gene cleavage or translation inhibition, function as traps for miRNA binding, or are directly targeted by miRNA to attenuate miRNA presence in plant immunity (Song et al., 2021). For instance, lncRNA *MuLnc1* was identified to be cleaved by *mul-miR3954* to produce *si16157* and negatively regulated *Botrytis cinerea* and *Pseudomonas syringae* resistance by inhibiting the functions of *calmodulin-like protein 27* (*CML27*) in mulberry (Gai et al., 2018). In tomato, *lncRNA42705* and *lncRNA08711* increase the expression levels of MYB genes by acting as decoys for *miR159* and enhance resistance against *P. infestans* (Cui et al., 2019); and, *Sl-lncRNA15492* was targeted by *SI-miR482a* to maintain S*l-NBS-LRR1* at an appropriate expression level during the immune response to *P. infestans* (Jiang et al., 2020). In this study of citrus and HLB, we identified a set of high confidence lncRNAs that potentially serve as precursors, eTMs, and targets of miRNAs. Among them, it is noteworthy that five lncRNAs simultaneously serve as eTMs and targets of miRNA, and eleven lncRNAs simultaneously serve as precursors and miRNA targets (**Table S8**). Notably, nine DE lncRNAs were specifically identified in sweet orange. Several miRNAs that interacted with these lncRNAs have been reported to be involved in disease resistance, such as *miR858* negatively regulated *Arabidopsis* immunity (Camargo-Ramírez et al., 2018), *miR477* enhanced the susceptibility of the tea plants to *Pseudopestalotiopsis* species infection (Wang et al., 2020), and *miRNA482* suppressed the expression of NBS-LRR defense genes in cotton (Zhu et al., 2014). Interestingly, *LNC_40405* and *LNC_69103* were predicted to act as eTMs of *miR477* and *miR482*, respectively, and were significantly downregulated in *C*Las-inoculated sweet orange at 7 WAI and 17 WAI. In addition, multiple DE lncRNAs were also targeted by *miR2111* and *miR5021*. A recent study indicated that *miR2111* positively regulates shoot-to-root systemic effectors of rhizobia and promotes nodule formation (Moreau et al., 2021). Surprisingly, *miR5021* was also predicted to be one having maximal matches against 19910 ESTs from periwinkle (*Catharanthus roseus*) (Pani and Mahapatra, 2013), which is an alternate host of the HLB bacteria (Zhang et al., 2010). Most of the predicted *miR5021* targets were the genes involved in cell growth and development, signaling, and metabolism in periwinkle. In citrus, we also found multiple genes associated with disease response were targeted by *miR5021* (**Table S12**). *LNC_69103* was not only acting as an eTM of *miR482* but also acting as a target of *miR5021*. Compared to healthy plants, *LNC_69103* was extremely downregulated (log2FC < -9) in *C*Las-infected sweet orange plants. These findings indicated the expression level of these lncRNAs might be tightly related to the sensitivity of citrus plants to HLB.

### 4.4 A potential regulatory model of *LNC_28805* in response to CLas

One of the main objectives of this study was to understand the expression dynamics and co-expression networks responding to citrus HLB in tolerant rough lemon and sensitive sweet orange. The WGCNA co-expression network revealed inoculation and temporal specific modules and important hub genes involved in citrus HLB response. Brown module was a unique significant module associated with *C*Las-inoculation in rough lemon and included a large group of co-expressed lncRNAs. Cluster analysis of expression pattern showed that lncRNAs displayed more temporal specific expression patterns than mRNAs and were significantly upregulated at 7 WAI (**Figure 4E**), suggesting that the functions of these lncRNAs might be closely related to HLB response at early stages after *C*Las-inoculation. We speculate that HLB-tolerant genes in rough lemon might be mediated by these early response lncRNAs. Evidence from most disease response genes exhibiting strong protein-protein interactions was identified in the blue module, but not the brown module, and these genes were significantly upregulated at 17 WAI, though not at 7 WAI (**Figure S11** and **Figure 6E**). Functional enrichment analysis indicated that co-expressed genes in rough lemon were mainly responsible for the response to reactive oxygen species, programmed cell death, and plant pathogen, which are highly related to disease defense (**Figure 6E**). Although genes in response to bacteria were also enriched in sweet orange, most of them were responsible for growth and development, fatty acids biosynthesis, and reproductive processes (**Figure 5B**). A fraction of genes was even associated with negative regulation of response to stimulus in sweet orange. These results also reflect the greater sensitivity of sweet orange to HLB than rough lemon.

Additionally, MapMan analysis identified multiple genes involved in pathogenic effector-triggered immunity, systemic acquired resistance, WRKY33-dependent plant immunity, and callose synthase. Their first neighboring genes in the co-expression network were highly correlated with pathogen response and exhibited strong interactions with each other. Interestingly, *WRKY33* and *SYP121* were two key hub genes with the highest edge weight in the PPI network (**Figure 6A**), and both of them are located in significant QTLs identified by the previous study (Huang et al., 2018), suggesting that these two genes might play important roles in HLB regulation. In addition, we found a hub *LNC_28805* was the most probable lncRNA involved in HLB regulation, which was co-expressed with multiple genes associated with the effector-triggered immunity (ETI) network, including *RIN4*, *CBP60*, *SIB*, and *NLR*. Moreover, *LNC_28805* has 145 first neighboring DE mRNAs (including *WRKY33* and *SYP121*) in the co-expression network. These neighboring genes were the most significantly enriched in the plant defense component (**Figure 6D**). Meanwhile, we also found that *LNC_28805*, belonging to intergenic lncRNAs type, was predicted to be targeted by the homologous *miR5021* of *Arabidopsis* with one mismatch, indicating that *LNC_28805* may be an evolutionarily conserved and functionally maintained lncRNA. We notably found that multiple disease resistance genes were targeted by *miR5021*, such as *WRKY33*, *SYP121*, and *NB-ARC* domain-containing disease resistance genes (**Table S12**), suggesting *LNC28805* might compete with endogenous *miR5021* to maintain the homeostasis of expression levels between immune-related genes and growth genes. All these results further indicate that *LNC28805* might be an important regulator involved in the HLB tolerance of rough lemon.

Plant WRKY transcription factors (TF) play key roles in plant responses to microbial infection. The PPI network showed WRKY33 was involved in multiple disease response pathways (**Figure 6A** and **Figure S14**). WRKY33 can regulate the expression of defense-related genes toward the necrotrophic fungus *B. cinerea*, but WRKY33 has also been shown to regulate cross-talk between jasmonate-, abscisic acid (ABA)-, and salicylic acid (SA)-regulated disease response pathways (Zheng et al., 2006; Birkenbihl et al., 2012; Liu et al., 2015). In *Arabidopsis*, the ectopic expression of *WRKY33* results in enhanced susceptibility to the bacterial pathogen *P. syringae* caused by the reduced expression of the salicylate-regulated PR-1 gene (Zheng et al., 2006). Loss of WRKY33 function results in activation of the ABA- and salicylic acid (SA)-related host response (Birkenbihl et al., 2012; Liu et al., 2015). The evidence indicated that high expression *WRKY33* might suppress the expression of ABA- or SA-regulated genes response to HLB bacteria (**Figure 7B**). WRKY33 also can activate the expression of RING-type ubiquitin ligase *ATL31* involved in vesicle trafficking with PEN1/SYP121 SNARE protein (Reyes et al., 2015), which function to guard cell membrane transport and stomatal control (Eisenach et al., 2012). Some others candidate genes in the PPI networks, such as *ACS6*, *ERF-1*, *CML37*, *CALS1*, *NSL1*, and *MPK1* involved in bacteria-induced ethylene, ABA, and JA signaling, callose synthesis, and SA-related defense (Tsuda and Katagiri, 2010; Luna et al., 2010; Fukunaga et al., 2017), were also putatively involved the defense response of *C*Las invasion (**Figures 6A** and **7B**). Based on the regulatory relationship of the genes in the networks, we suggested that *LNC_28805* may play an important role in maintaining the homeostasis of antagonistic relationship between defense pathways mediating WRKY33 associated with ABA- or SA-regulated genes involved *C*Las response (Zheng et al., 2006; Liu et al., 2015).

In addition, WRKY33 also can activate the expression of *Arabidopsis* RING-type ubiquitin ligase *ATL31* involved in vesicle trafficking with PEN1/SYP121 SNARE protein (Reyes et al., 2015), which functions to guard cell membrane transport and stomatal control (Eisenach et al., 2012). Because *LNC28805* and some of its co-expressed genes were targeted by *miRNA5021*, we suggest that *LNC28805* is probably involved in regulating the expression level of pathogenic response genes by competing for endogenous *miR5021*. If this hypothetical mechanism for HLB tolerance is correct, it should also be observed in plants grown in the field. The expression levels of *miR5021* targets (*WRKY33*, *SYP121*, *LNC28805*) presented significantly higher in rough lemon than in sweet orange under HLB stress for more than ten years in the field (**Figure 7C**). However, the expression levels of *miRNA5021* were reversed between them. It indicates that this relationship also exists in the naturally infected plants grown in the field. Thus, we suggest that *miR5021* targeting *WRKY33* and *SYP121* might promote the expression of genes responding to *C*Las. Dynamic expression of *WRKY33* might be required to balance the expression levels between immune-related genes and growth genes. *LNC28805* probably plays an important role in regulating the expression level of these pathogenic response genes by competing for endogenous *miR5021*.

Though callose deposition plays a role in defense against the pathogen, overaccumulation of callose inhibits phloem transport activities in *C*las-infected citrus (Achor et al., 2010). According to the previous study, callose-plugged phloem sieve elements were less serious in HLB-diseased rough lemon than in HLB-diseased sweet orange (Fan et al., 2012). A potential possibility of HLB tolerance mechanisms of rough lemon might be that *LNC28805* is involved in competing for endogenous *miR5021* to promote the expression of *WRKY33* and *SYP121*, which might function to suppress the immune-related genes overresponse to *C*Las infection and enhance the activity of phloem transport by reducing callose deposition (Fan et al., 2012; Deng et al., 2019). Taken together, our results not only represent the gene modules of lncRNAs and mRNAs related to pathogenic response but also bring new insights into the roles of lncRNAs acting as potential regulatory factors for citrus HLB tolerance.

## 5 Conclusion

To conclude, we systematically identified and characterized 8,742 lncRNAs among HLB-tolerant rough lemon and HLB-sensitive sweet orange from different time points after *C*Las-inoculation. Based on the integrated analysis of sequence conservation and variation, spatiotemporal-specific expression, functional enrichment, and lncRNA-mRNA co-expression networks with WGCNA, we identified a fraction of lncRNAs and mRNAs that were potentially responsive to *C*Las bacterium infection in citrus. *LNC_28805* was identified as one of the most important candidate lncRNAs involved in citrus HLB regulation. Two key candidates (*WRKY33* and *SYP121*) in the PPI network are known to negatively regulate bacteria pathogen responses and were found within overlapping QTLs identified in our previous study. Based on the reported studies and PPI network and gene co-expression networks in this study, a putative hypothesis for the regulatory pathway of *LNC_28805* is proposed (**Figure 7B**). This study will be useful in understanding the role of lncRNAs involved in citrus HLB regulation and provide a foundation for further investigation of their regulatory functions.

## Data availability statement

The datasets presented in this study can be found in online repositories. The names of the repository/repositories and accession number(s) can be found in the article/**Supplementary Material**. The raw data of RNA sequencing presented in the study are deposited in the NCBI repository, GEO accession number is GSE215306.

## Author contributions

Conceptualization, XZ, QY, and FG; Data curation, XZ, QY, and RR; Methodology, XZ, QY, RR, YZ, XW, PH, and YW; Formal analysis, XZ, QY, and RR; Funding acquisition, FG; Investigation, XZ, QY, YZ, RR, and XW; Software and visualization, XZ and RR; Writing—original draft, XZ; and Writing—review and editing, XZ, QY, PH, RR, YW, and FG. All authors contributed to the article and approved the submitted version.
