# Supplementary material for: Role of long non-coding RNA in regulatory network response to Candidatus Liberibacter asiaticus in citrus
Source: Front Plant Sci. 2023 Feb 20;14:1090711. doi: 10.3389/fpls.2023.1090711 (PMC9986497; doi:10.3389/fpls.2023.1090711)
Supplement: Supplementary file 1 [file DataSheet_1.docx]

**Supplementary figure legend**

**Figure S1** Overall characteristics of lncRNAs in citrus.

**Figure S2** The correlations of expression profiles of lncRNAs.

**Figure S3** Distribution patterns of exon number and length of **(a)** lncRNAs and **(b)** protein-coding mRNAs in rough lemon and sweet orange.

**Figure S4** Principal component analysis (PCA) showing global relationships between replicates and mock- or *C*Las-inoculated and time course samples.

**Figure S5** Validation of RNA-seq data via qRT-PCR.

**Figure S6** Venn diagram representing the number of common and specific differentially expressed (DE) lncRNAs in different pairwise compared groups.

**Figure S7** Identification of similar expression patterns lncRNA and mRNAs between healthy and HLB-affected plants of rough lemon.

**Figure S8** Identification of similar expression patterns lncRNA and mRNAs between healthy and HLB-affected plants of sweet orange.

**Figure S9** Hierarchical clustering and lncRNA-mRNA co-expression modules in sweet orange.

**Figure S10** A scatterplot showing the relationship between gene significance for weeks after inoculation (WAI) and module membership in magenta, purple, yellow, turquoise, blue, green, and pink modules.

**Figure 11** Heatmap showing the normalized FPKM (NFPKM) of mRNA in each significant module in **(a)** rough lemon and **(b)** sweet orange. FPKM were normalized to the Z-score.

**Figure S12** GO and MapMan enrichment of genes in the significant WGCNA modules in rough lemon and sweet orange.

**Figure S13** The co-expression network of genes first neighboring with pathogen response and callose synthase related genes.

**Figure S14** Interaction relationship of co-expression genes in rough lemon and sweet orange.

**Figure S15** Plants of rough lemon and sweet orange under HLB stress more than ten years in the field.


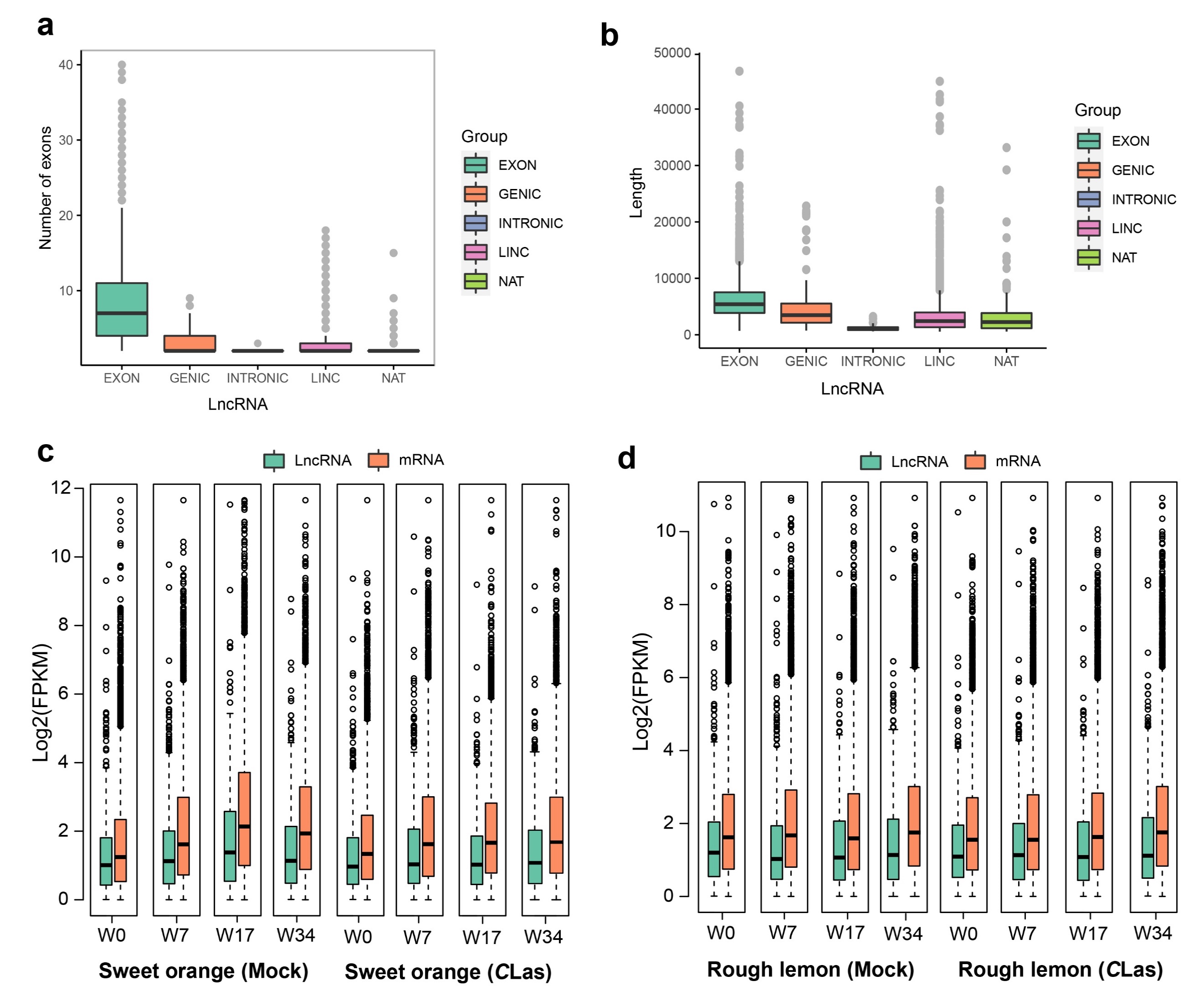


**D**

**C**

**B**

**A**

**Figure S1** Overall characteristics of lncRNAs in citrus. **(A)** The exon numbers and **(B)** length distributions of intergenic lncRNAs (LINC), intronic lncRNA (INTRONIC), natural antisense transcripts (NAT), genic lncRNA (GENIC), and exonic lncRNA (EXON). **(C-D)** Comparison of expression levels of lncRNAs and mRNAs across four different time points after mock- and CLas-inoculation in **(C)** sweet orange and **(D)** rough lemon.


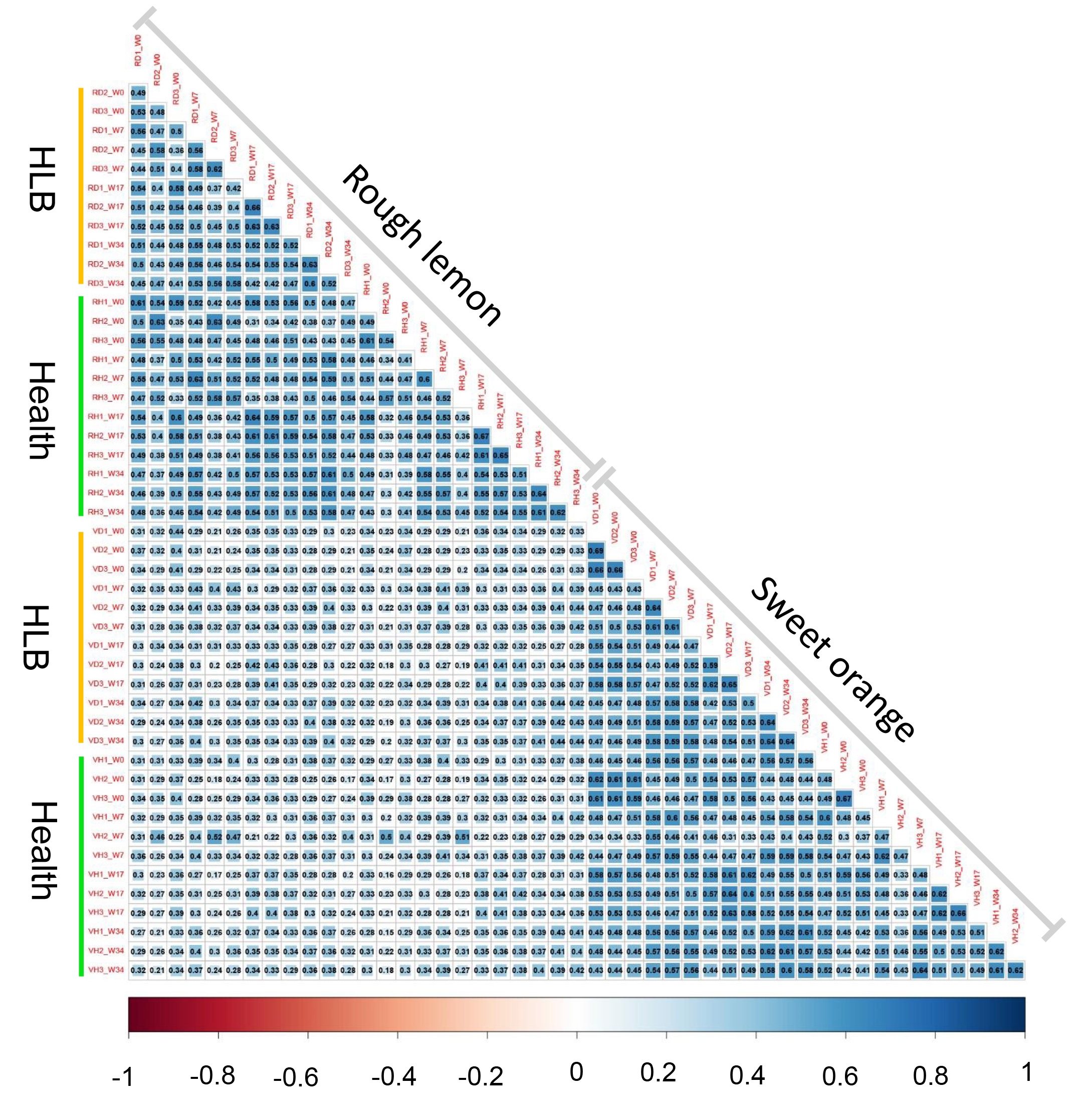


**Figure S2** The correlations of expression profiles of lncRNAs.


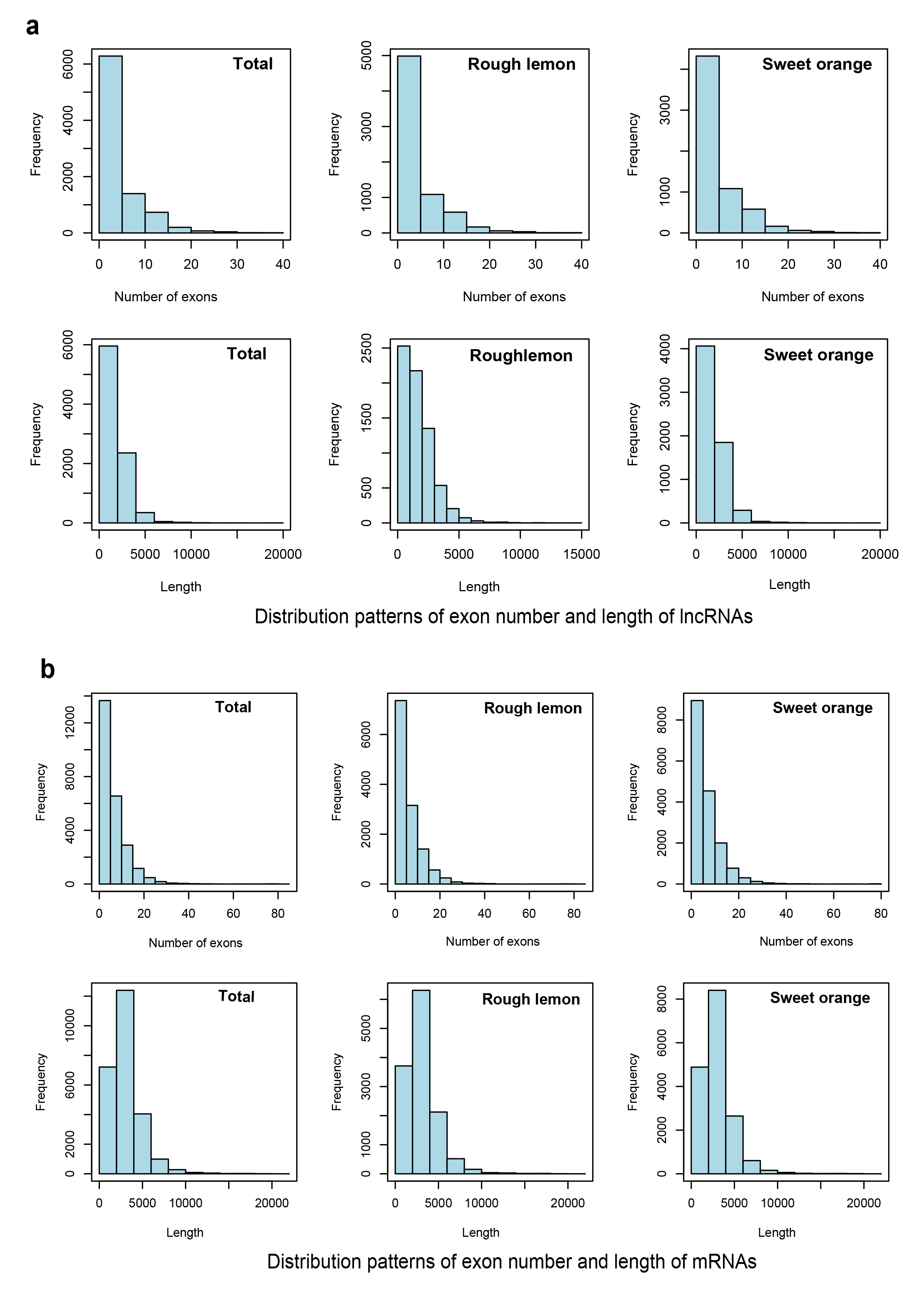


**A**

**B**

**Figure S3** Distribution patterns of exon number and length of **(A)** lncRNAs and **(B)** protein-coding mRNAs in rough lemon and sweet orange. **(A)** The distribution patterns of exon number and length of lncRNAs in rough and sweet orange. **(B)** The distribution patterns of exon number and length of mRNAs. The “total” indicates exon number or length of lncRNA or mRNA in both rough lemon and sweet orange.


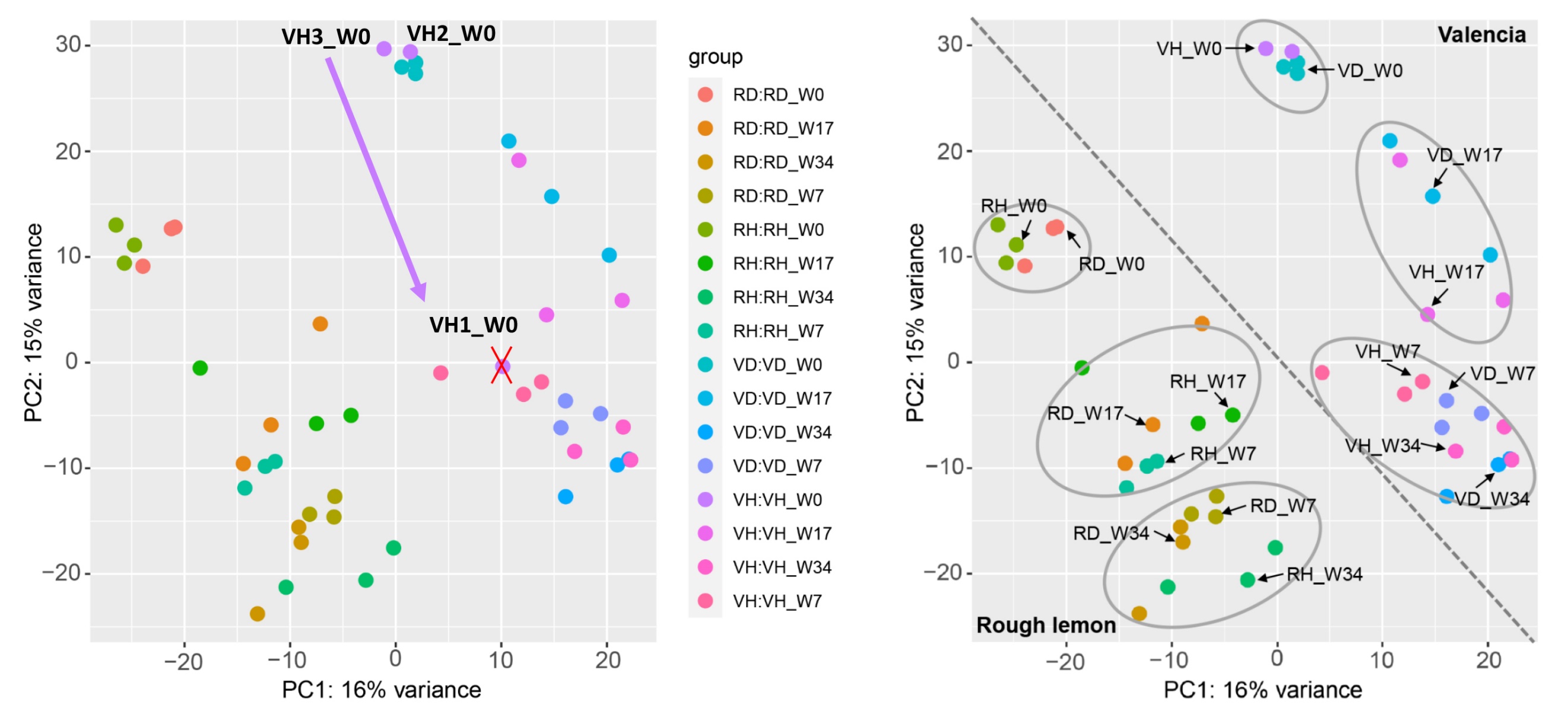


**Figure S4** Principal component analysis (PCA) showing global relationships between replicates and mock- or *C*Las-inoculated and time course samples. VH1_W0 samples from VH:VH_W0 group seriously deviated from the other two replicates and was removed in the analysis.


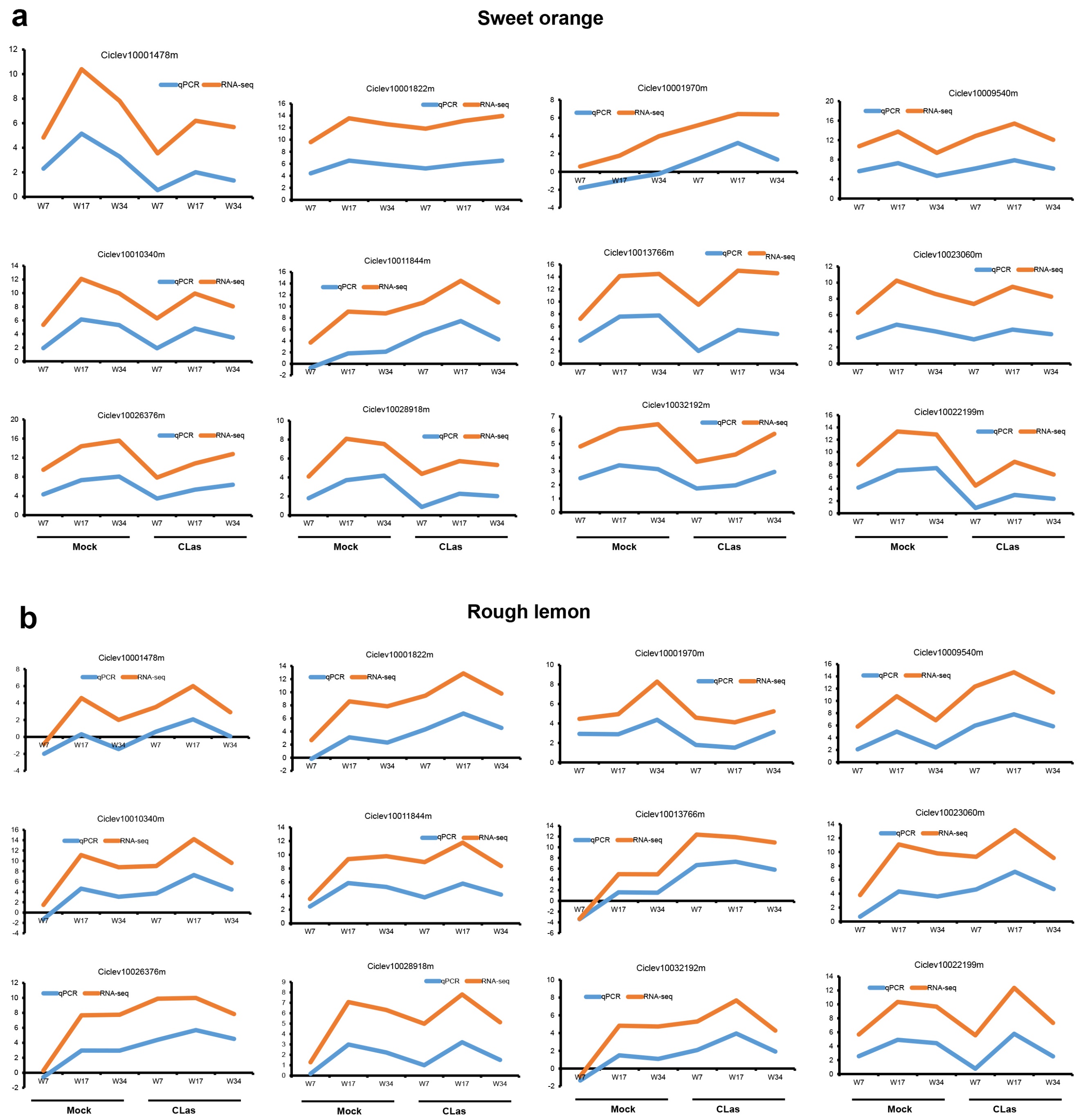


**B**

**A**

**Figure S5** Validation of RNA-seq data via qRT-PCR. **(A)** Relative expression of genes in sweet orange. **(B)** Relative expression of genes in rough lemon.


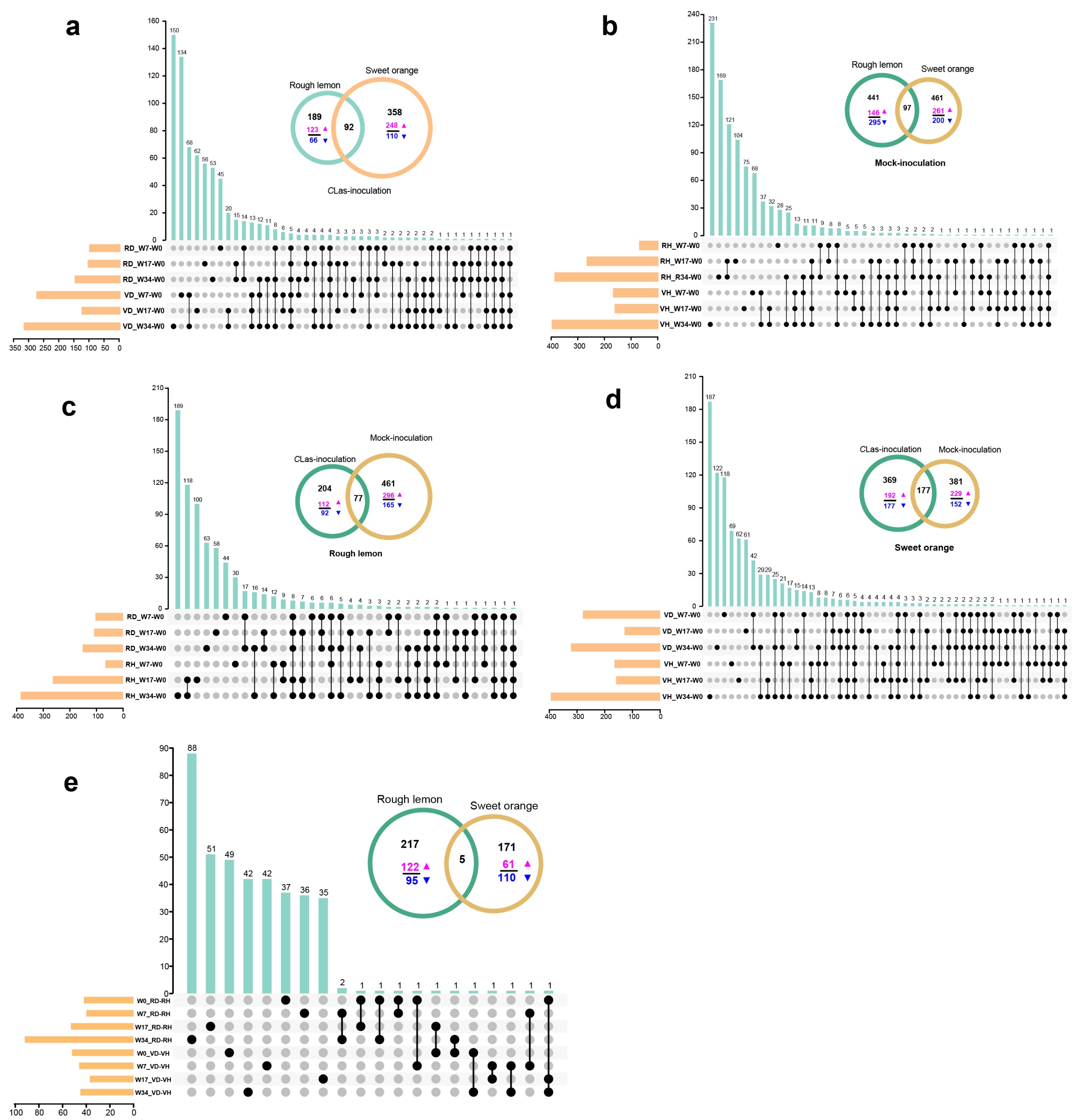


**E**

**D**

**C**

**B**

**A**

**Figure S6** Venn diagram representing the number of common and specific differentially expressed (DE) lncRNAs in different pairwise compared groups. **(A-B)** Number of common and specific in W7-W0, W17-W0, and W34-W0 pairwise groups of **(A)** *C*Las-inoculated and **(B)** mock-inoculated plants between the comparison of rough lemon and sweet orange. (**C-D**) Number of common and specific in W7-W0, W17-W0, and W34-W0 pairwise groups between the comparison of mock- and *C*Las-inoculated plants in **(C**) rough lemon and **(D)** sweet orange. **(E)** Number of specifically expressed lncRNAs between mock- and *C*Las-inoculation pairwise groups at W0, W7, W17, and W34 stages. The pink and blue triangles indicate up- and down-regulation, respectively.


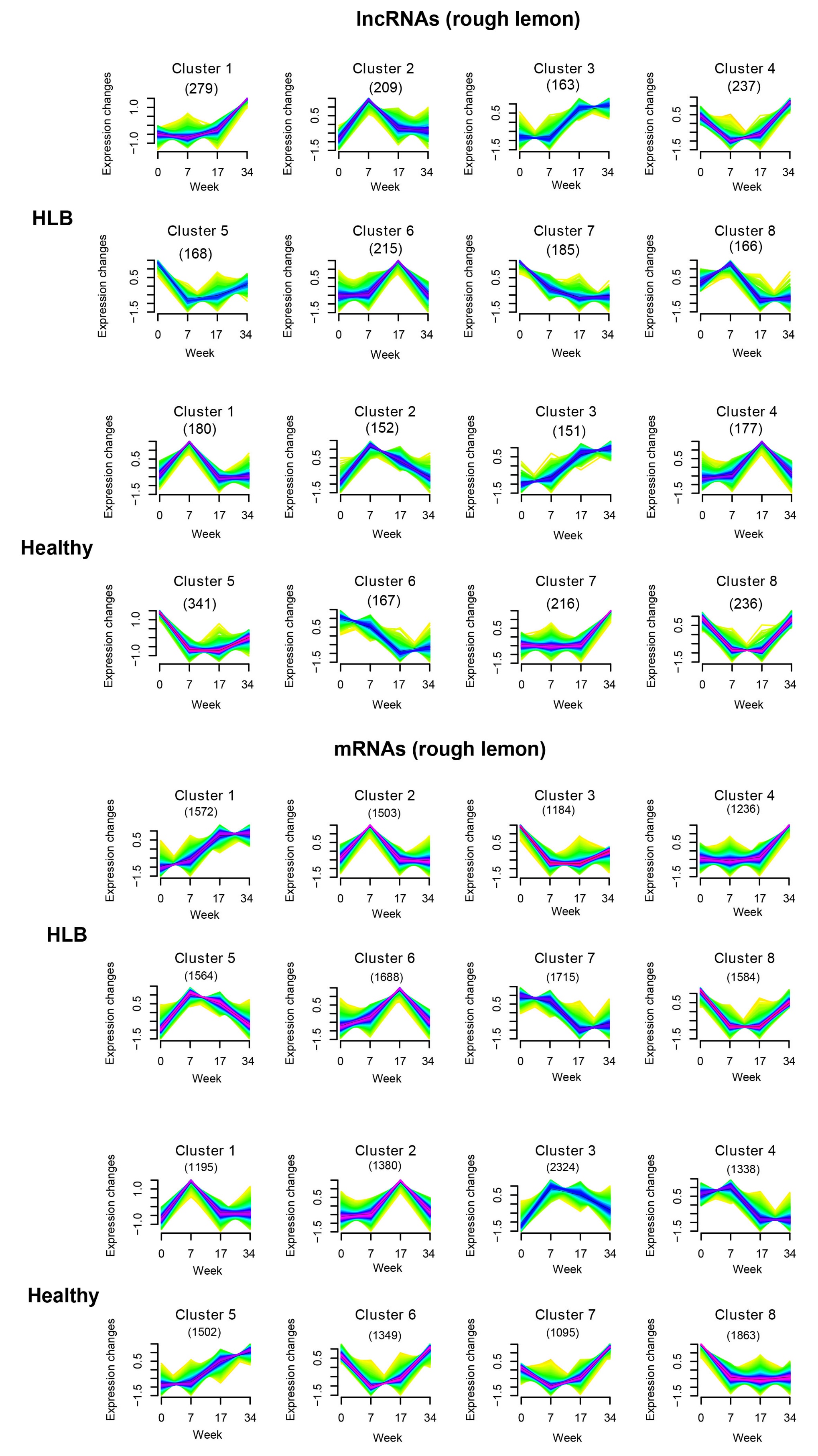


**Figure S7** Identification of similar expression patterns of lncRNAs and mRNAs between healthy and HLB- affected plants in rough lemon.


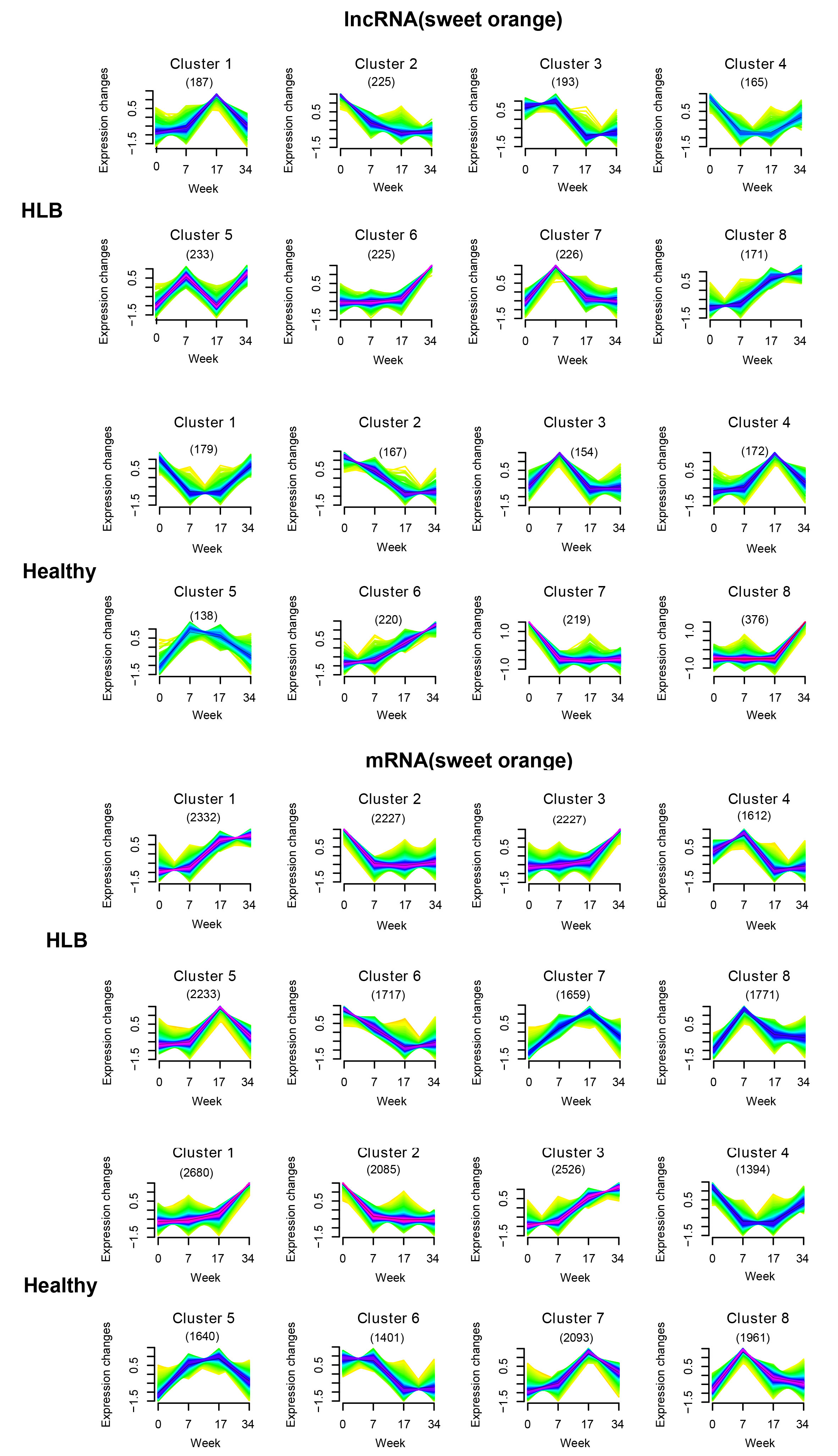


**Figure S8** Identification of similar expression patterns lncRNA and mRNAs between healthy and HLB-affected plants in sweet orange.


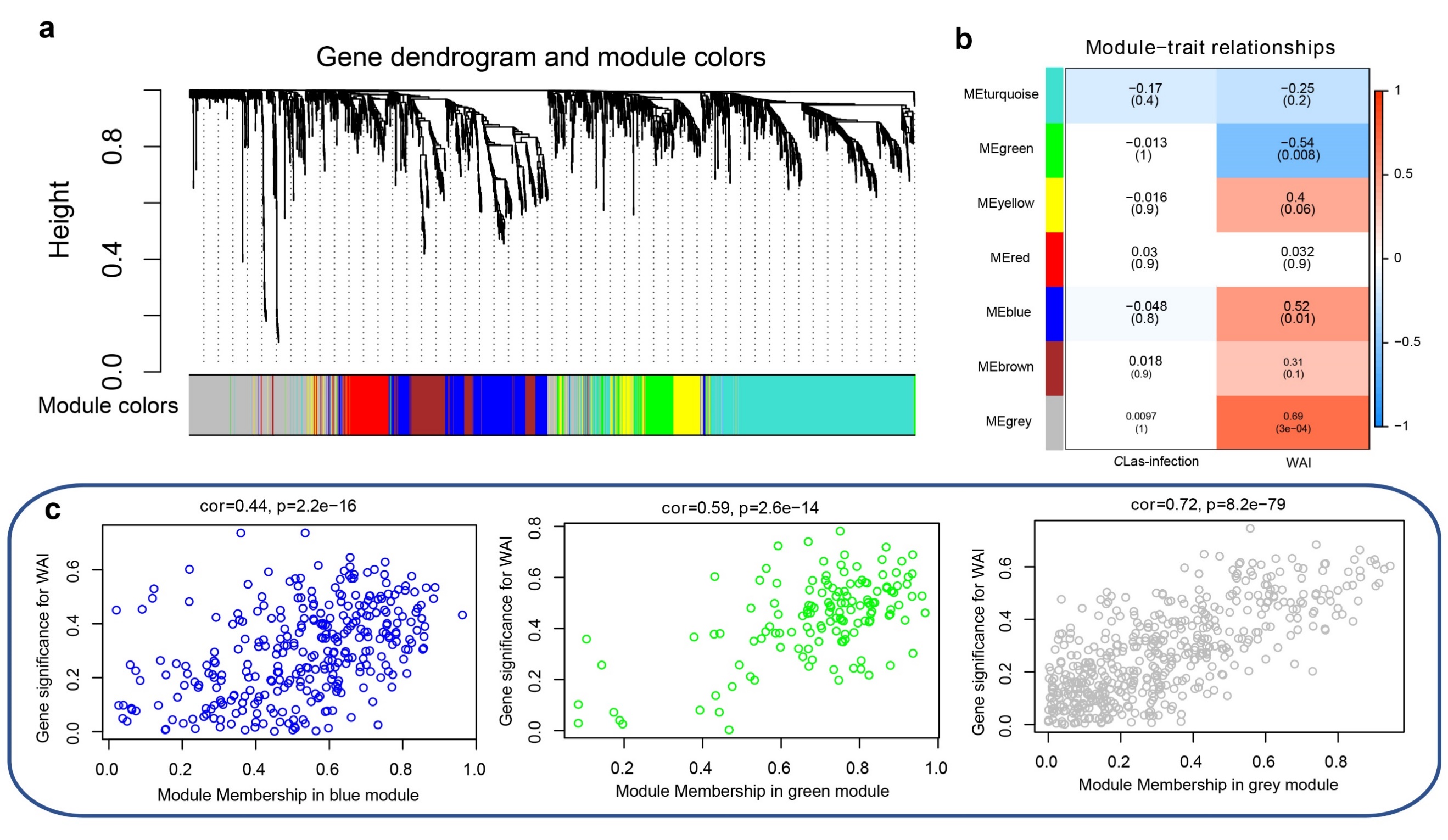


**C**

**B**

**A**

**Figure S9** Hierarchical clustering and lncRNA-mRNA co-expression modules in sweet orange. **(A)** Hierarchical cluster tree showing gene co-expression modules identified by Weighted Gene Co-expression Network Analysis (WGCNA). Seven different modules were constructed and labeled by different colors. **(B)** Module-inoculation or module-WAI (weeks after inoculation) relationship. Each row corresponds to a module; left column corresponds to inoculation approach and right column corresponds to the time after inoculation. Each cell is color-coded by correlation coefficient and contains corresponding *P*-value. **(C)** A scatterplot showing the relationship between gene significance for WAI and module membership in blue, green, and grey modules.


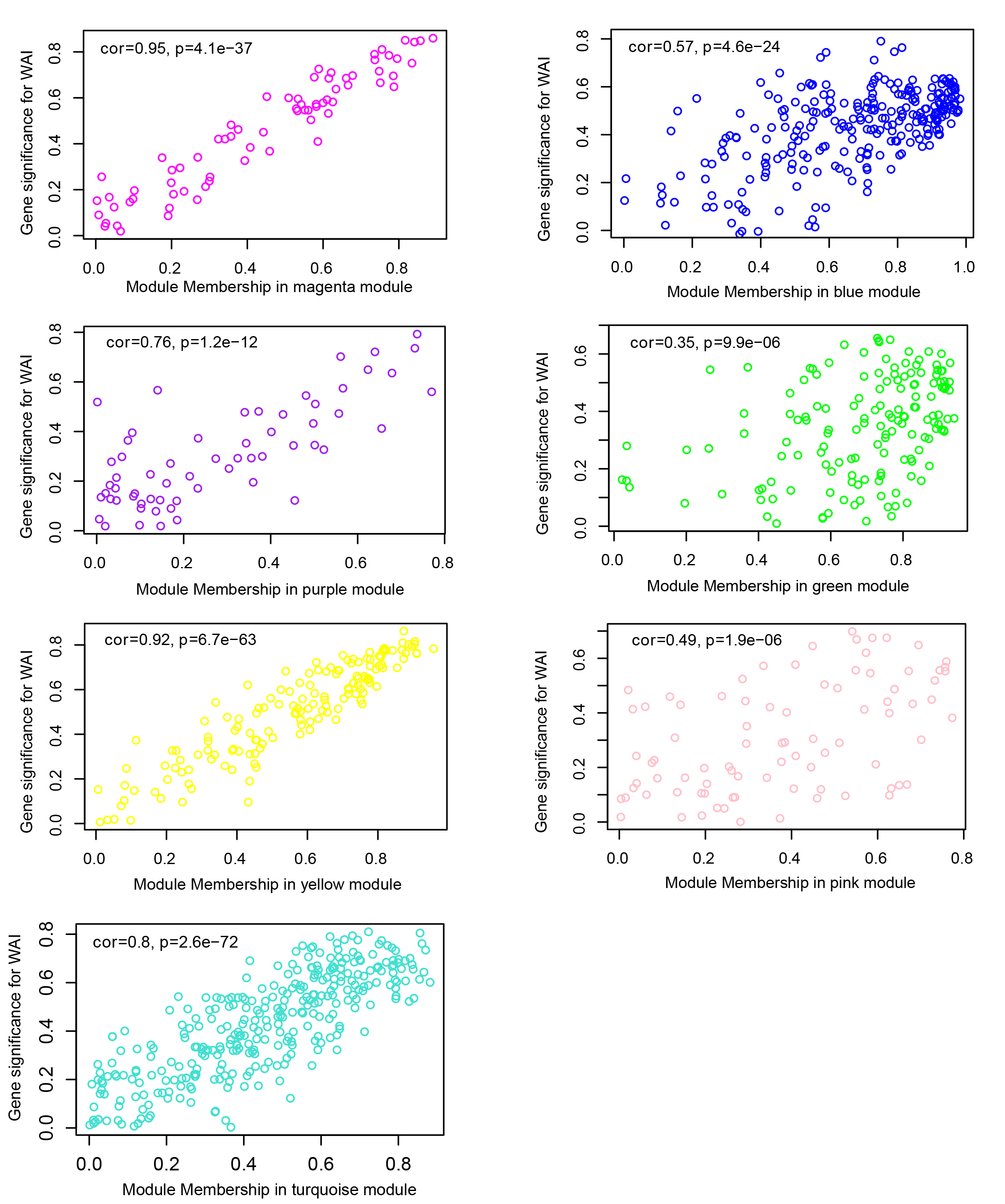


**Figure S10** A scatterplot showing the relationship between gene significance for weeks after inoculation (WAI) and module membership in magenta, purple, yellow, turquoise, blue, green, and pink modules.


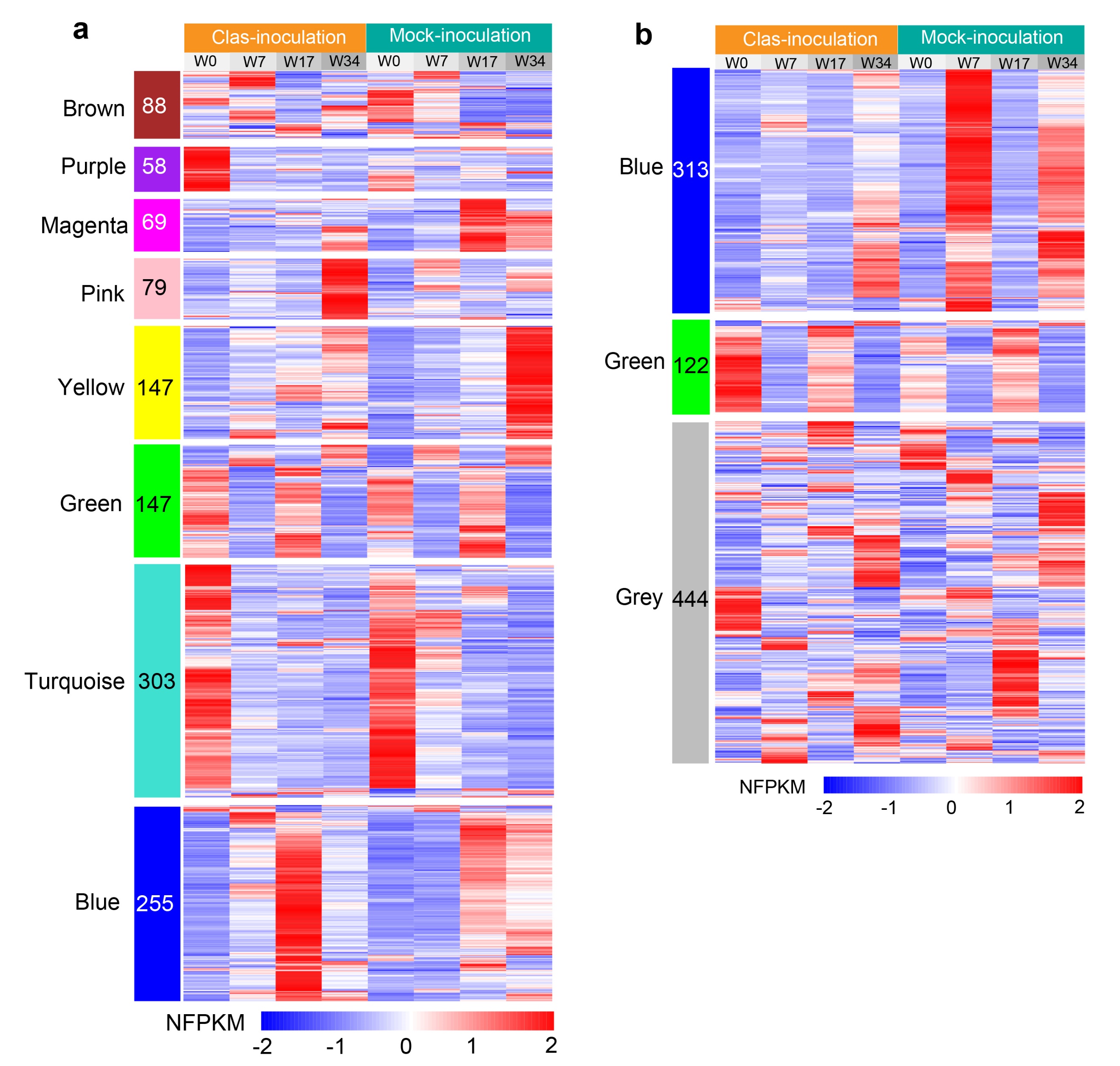


**B**

**A**

**Figure 11** Heatmap showing the normalized FPKM (NFPKM) of mRNA in each significant module in **(A)** rough lemon and **(B)** sweet orange. FPKM were normalized to the Z-score.


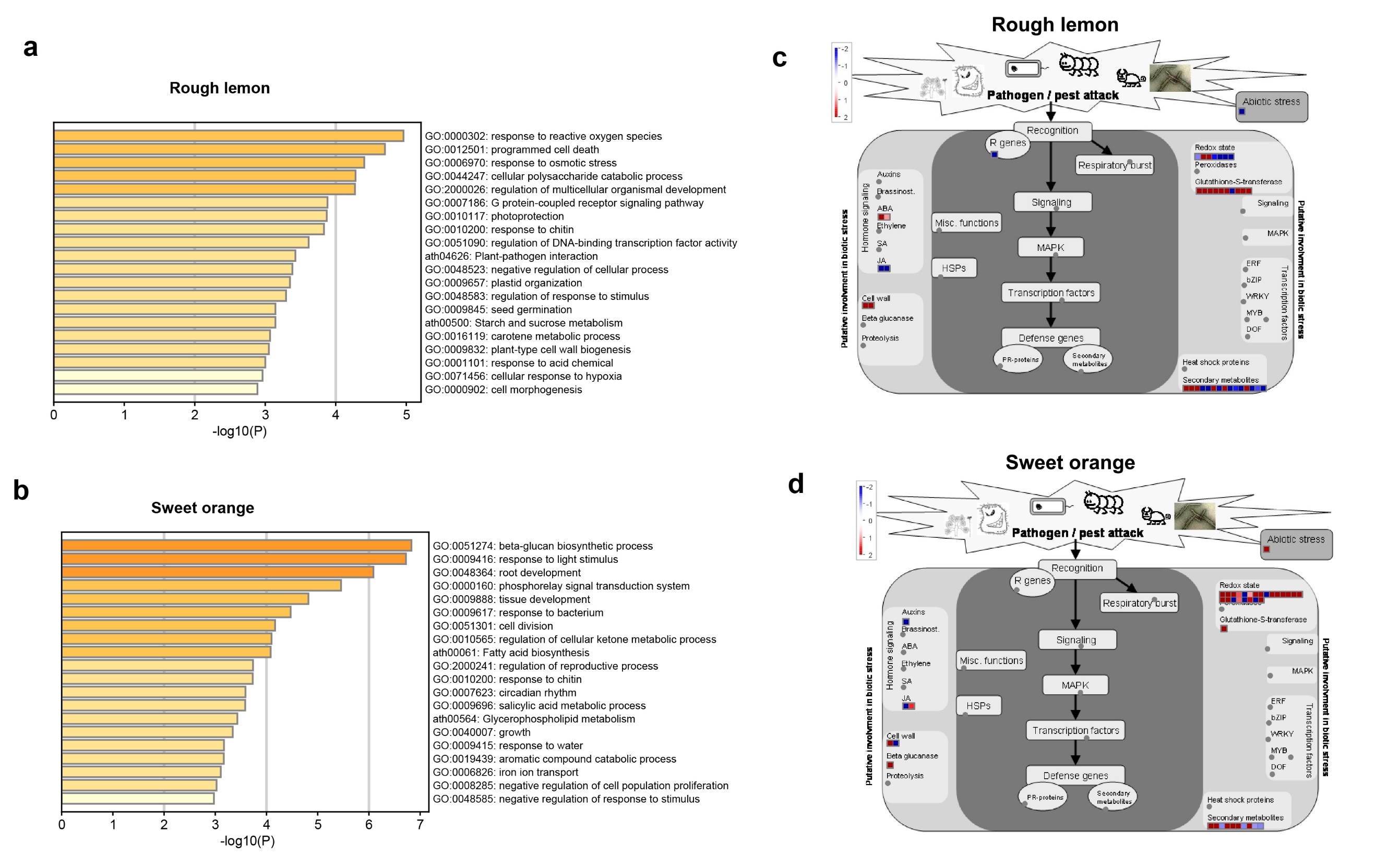


**D**

**C**

**B**

**A**

**Figure S12** GO and MapMan enrichment of genes in the significant WGCNA modules in rough lemon and sweet orange. **(A-B)** Top 20 clusters with representative enriched GO terms in **(A)** rough lemon and **(B)** sweet orange, colored by *P*-values. **(C-D)** MapMan analysis of biotic stress-related genes in the significant WGCNA modules in **(C)** rough lemon and **(D)** sweet orange. The biotic stress overview showed that redox state, glutathione-S-transferase, and secondary metabolites were highly enriched. Boxes with red and blue colors indicate up and downregulated genes, respectively.


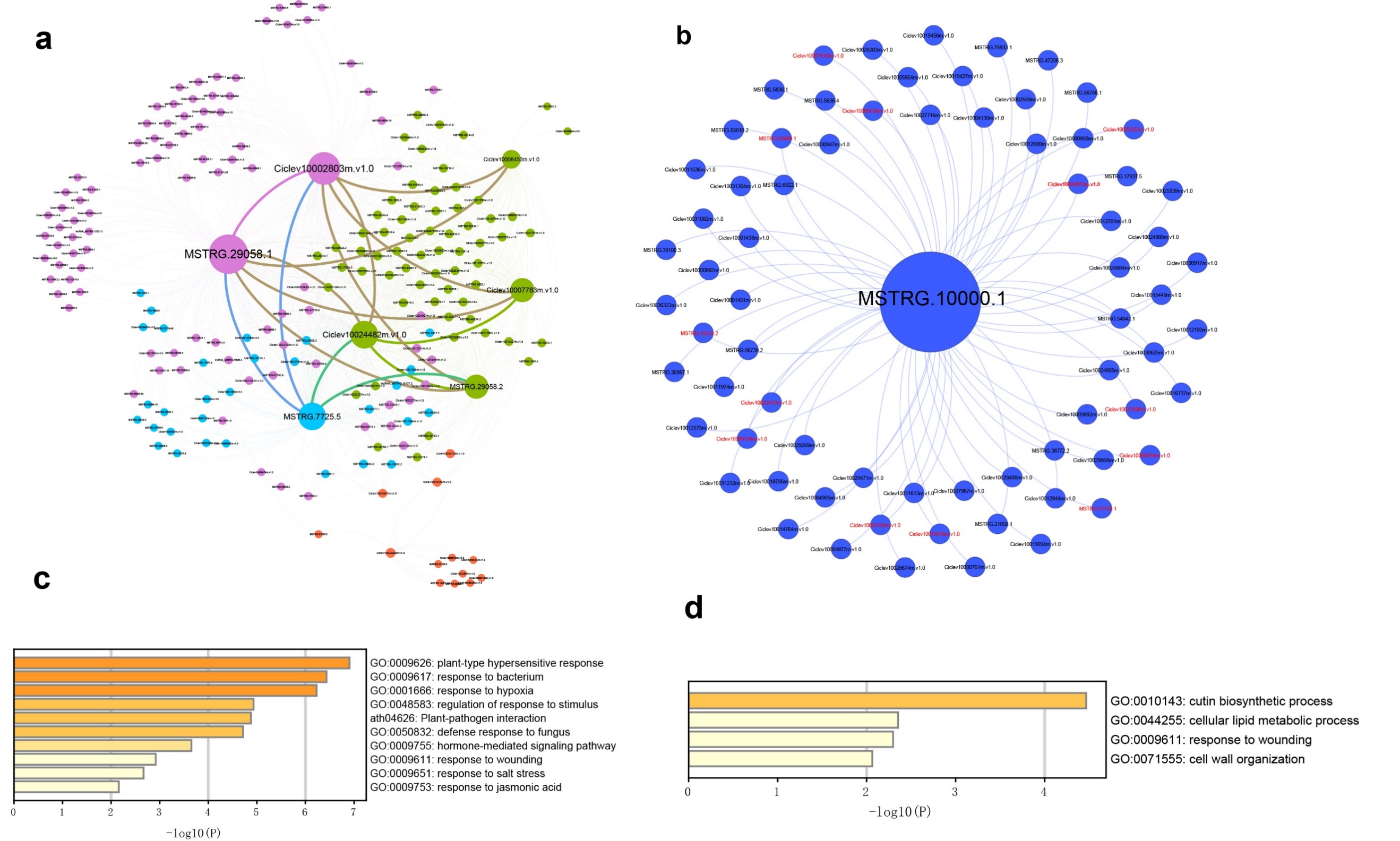


**A**

**D**

**C**

**B**

**Figure S13** The co-expression network of genes first neighboring with pathogen response and callose synthase related genes. **(A)** The first neighboring genes of pathogen response related genes with edge weight higher than 0.2 in rough lemon. **(B)** The first neighboring genes of callose synthase related genes with edge weight higher than 0.2 in sweet orange. **(C-D)** GO enrichment of genes in the network in **(C)** rough lemon and **(D)** sweet orange.


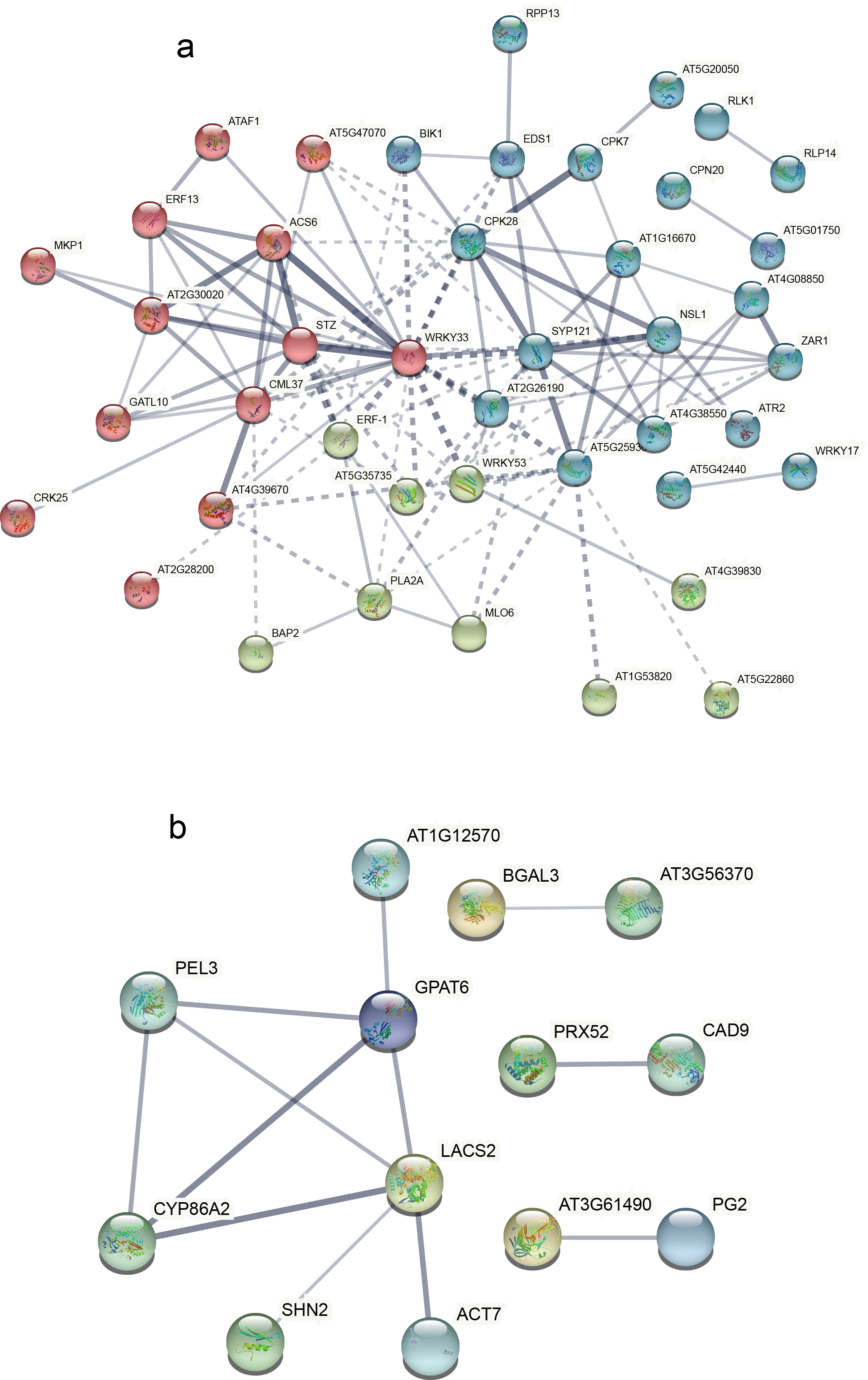


**B**

**A**

**Figure S14** Interaction relationship of co-expression genes in rough lemon and sweet orange. **(A)** PPI network of the co-expressed genes related to pathogen response in rough lemon. **(B)** PPI network of the co-expressed genes related to callose synthase in sweet orange. The edge confidence of interacted genes is higher than 0.4. Proteins of *A. thaliana* were used as model to infer the PPI network of the co-expressed genes in citrus species.


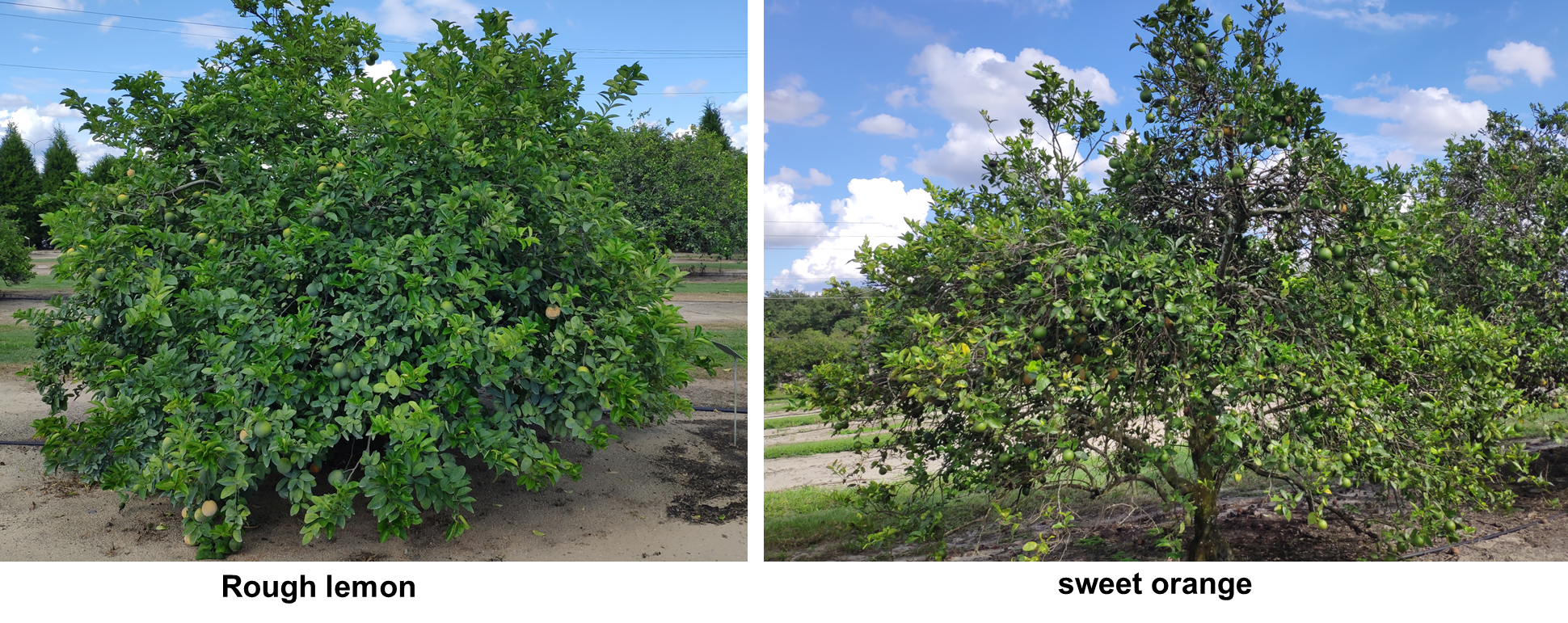


**Figure S15** Plants of rough lemon and sweet orange under HLB stress more than ten years in the field.
